# Observation of a correlated free four-neutron system

**DOI:** 10.1038/s41586-022-04827-6

**Published:** 2022-06-22

**Authors:** M. Duer, T. Aumann, R. Gernhäuser, V. Panin, S. Paschalis, D. M. Rossi, N. L. Achouri, D. Ahn, H. Baba, C. A. Bertulani, M. Böhmer, K. Boretzky, C. Caesar, N. Chiga, A. Corsi, D. Cortina-Gil, C. A. Douma, F. Dufter, Z. Elekes, J. Feng, B. Fernández-Domínguez, U. Forsberg, N. Fukuda, I. Gasparic, Z. Ge, J. M. Gheller, J. Gibelin, A. Gillibert, K. I. Hahn, Z. Halász, M. N. Harakeh, A. Hirayama, M. Holl, N. Inabe, T. Isobe, J. Kahlbow, N. Kalantar-Nayestanaki, D. Kim, S. Kim, T. Kobayashi, Y. Kondo, D. Körper, P. Koseoglou, Y. Kubota, I. Kuti, P. J. Li, C. Lehr, S. Lindberg, Y. Liu, F. M. Marqués, S. Masuoka, M. Matsumoto, J. Mayer, K. Miki, B. Monteagudo, T. Nakamura, T. Nilsson, A. Obertelli, N. A. Orr, H. Otsu, S. Y. Park, M. Parlog, P. M. Potlog, S. Reichert, A. Revel, A. T. Saito, M. Sasano, H. Scheit, F. Schindler, S. Shimoura, H. Simon, L. Stuhl, H. Suzuki, D. Symochko, H. Takeda, J. Tanaka, Y. Togano, T. Tomai, H. T. Törnqvist, J. Tscheuschner, T. Uesaka, V. Wagner, H. Yamada, B. Yang, L. Yang, Z. H. Yang, M. Yasuda, K. Yoneda, L. Zanetti, J. Zenihiro, M. V. Zhukov

**Affiliations:** 1 Technische Universität Darmstadt, Fachbereich Physik, Darmstadt, Germany; 2 GSI Helmholtzzentrum für Schwerionenforschung GmbH, Darmstadt, Germany; 3 Helmholtz Forschungsakademie Hessen für FAIR, Frankfurt, Germany; 4 Technische Universität München, Physik Department, Garching, Germany; 5 RIKEN Nishina Center for Accelerator-Based Science, Wako, Japan; 6 Department of Physics, University of York, York, UK; 7 LPC-Caen, IN2P3/CNRS, UniCaen, ENSICAEN, Normandie Université, Caen, France; 8 Department of Physics and Astronomy, Texas A&M University-Commerce, Commerce, TX USA; 9 IRFU, CEA, Université Paris-Saclay, Gif-sur-Yvette, France; 10 Dpt. de Física de Partículas, Universidade de Santiago de Compostela, Santiago de Compostela, Spain; 11 Nuclear Energy group, ESRIG, University of Groningen, Groningen, The Netherlands; 12 Atomki, Eötvös Loránd Research Network (ELKH), Debrecen, Hungary; 13 State Key Laboratory of Nuclear Physics and Technology, School of Physics, Peking University, Beijing, China; 14 Rudjer Bošković Institute, Zagreb, Croatia; 15 Ewha Womans University, Seoul, Korea; 16 Center for Exotic Nuclear Studies, Institute for Basic Science, Daejeon, Korea; 17 Department of Physics, Tokyo Institute of Technology, Tokyo, Japan; 18 Department of Physics, Tohoku University, Miyagi, Japan; 19 Department of Physics, The University of Hong Kong, Hong Kong, China; 20 Department of Physics, Chalmers University of Technology, Gothenburg, Sweden; 21 Center for Nuclear Study, The University of Tokyo, Tokyo, Japan; 22 Universität zu Köln, Institut für Kernphysik, Cologne, Germany; 23 Institute of Space Sciences, Magurele, Romania; 24 GANIL, CEA/DRF-CNRS/IN2P3, Caen, France; 25 Department of Physics, Kyoto University, Kyoto, Japan

**Keywords:** Experimental nuclear physics

## Abstract

A long-standing question in nuclear physics is whether chargeless nuclear systems can exist. To our knowledge, only neutron stars represent near-pure neutron systems, where neutrons are squeezed together by the gravitational force to very high densities. The experimental search for isolated multi-neutron systems has been an ongoing quest for several decades^1^, with a particular focus on the four-neutron system called the tetraneutron, resulting in only a few indications of its existence so far^2–4^, leaving the tetraneutron an elusive nuclear system for six decades. Here we report on the observation of a resonance-like structure near threshold in the four-neutron system that is consistent with a quasi-bound tetraneutron state existing for a very short time. The measured energy and width of this state provide a key benchmark for our understanding of the nuclear force. The use of an experimental approach based on a knockout reaction at large momentum transfer with a radioactive high-energy ^8^He beam was key.

## Abstract (web summary)

Experiment based on knocking out an alpha particle from a high-energy helium isotope shows a resonance-like structure that is consistent with a quasi-bound tetraneutron state existing for a very short time.

## Main

A neutron can be bound either in an atomic nucleus or in a neutron star. The free neutron has a lifetime of just under 15 min and decays into a proton, electron and antineutrino. The system made of two neutrons, the dineutron, is known to be unbound by only about 100 keV. Whether multi-neutron systems can exist as weakly bound states or very short-lived unbound resonant states has been a long-standing question^1^. The next simplest system of three neutrons is less likely to exist owing to the odd number of nucleons and therefore weaker binding; yet, a recent calculation has suggested its existence^5^. Following these considerations, the four-neutron system, the tetraneutron, is an appropriate candidate to address this question. An overview of previous experiments and theoretical approaches is given in ref. ^1^. 

Numerous attempts have been made to find a hint for the existence of the tetraneutron as a bound or resonant state. Among these attempts, experiments were performed searching for possible bound tetraneutrons produced in uranium fission reactions (see, for example, ref. ^6^). Other attempts, sensitive to both bound and resonant states, used pion-induced double-charge-exchange (DCX) reactions, mainly the ${}^{4}{\rm{H}}{\rm{e}}({{\rm{\pi }}}^{-},\,{{\rm{\pi }}}^{+})$ reaction (see, for example, ref. ^7^), as well as transfer reactions such as ${}^{8}{\rm{H}}{\rm{e}}({\rm{d}},{}^{6}{\rm{L}}{\rm{i}})$ (ref. ^8^). None of the experiments yielded a positive signal.

Most of the past experiments were performed with stable nuclei. Towards the twenty-first century, with the development of radioactive-ion beam facilities, it became possible to use extremely neutron-rich nuclei in which one can expect an enhanced formation of a tetraneutron system. The first indication for a possible bound tetraneutron was reported in 2002^2^ from a break-up reaction of ^14^Be into ^10^Be + ^4^n. The result stimulated several theoretical studies, all agreeing on the same conclusion: a bound tetraneutron state cannot be obtained theoretically without significantly changing our understanding of the nuclear forces^9–11^. However, the possibility of the four-neutron system existing as a resonant quasi-bound state with a very short lifetime on the order of a few 10^−22^ s, before decaying, has remained an open and challenging question. It was later found that the result reported in ref. ^2^ is also consistent with such a resonant state with the limit on its energy ${E}_{{\rm{r}}}\lesssim 2\,{\rm{MeV}}$ (ref. ^3^).

A decade later, in 2016, an indication of a tetraneutron resonance was reported^4^. A DCX reaction was used, but in contrast to previous attempts, this time the reaction was induced by a high-energy ^8^He radioactive beam. ^8^He is the most neutron-rich bound isotope, and the ^8^He(^4^He, ^8^Be) reaction channel was investigated. The advantage of using a radioactive beam is the freedom of selecting the reaction partner in a so-called recoil-less production (without momentum transfer) of the four-neutron system. The energy of the state was found to be *E*_r_ = 0.8 ± 1.4 MeV, and an upper limit on its width was estimated as *Γ* ≤  2.6 MeV. However, owing to the large experimental uncertainty, the possibility of a bound state could not be excluded by this experiment.

**Fig. 1:**
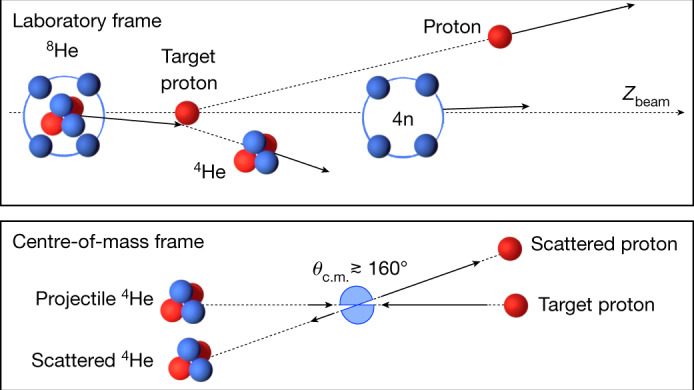
Schematic illustration of the quasi-elastic reaction investigated in this work. Top: quasi-elastic scattering of the ^4^He core from a ^8^He projectile off a proton target in the laboratory frame. The length of the arrows represents the momentum per nucleon (the velocity) of the incoming and outgoing particles. *Z*_beam_ is the beam axis. Bottom: the equivalent p–^4^He elastic scattering in their centre-of-mass frame, where we consider reactions at backward angles close to 180°, *θ*_c.m._ ≳ 160°. In this frame, the momentum of the proton balances that of the ^4^He, ${{\bf{P}}}_{{\rm{p}}}=-{{\bf{P}}}_{{}^{4}{\rm{He}}}$, that is, the proton is four times faster than the ^4^He.

In this work, we used the quasi-elastic knockout of an α-particle (^4^He nucleus) from a high-energy ^8^He projectile induced by a proton target to populate a possible tetraneutron state. The inverse-kinematics knockout reaction ${}^{8}{\rm{He}}({\rm{p}},\,{{\rm{p}}}^{4}{\rm{He}})$ at large momentum transfer is well suited because the ^8^He nucleus has the pronounced cluster structure of an α-core (^4^He) and four valence neutrons with small 4n centre-of-mass motion, such that after the sudden removal of the α-particle, a rather localized four-neutron system with small relative energy between the neutrons is produced, which may have a large overlap with a tetraneutron state^12,13^. The chosen kinematics at large momentum transfer between the proton and the α-particle ensures that the four-neutron system will recoil only with the intrinsic momentum of the ^4^He core in the ^8^He rest frame, without any further momentum transfer, thus allowing the recoil-less production. Furthermore, final-state interactions between the four neutrons and the charged particles are also minimized owing to the large momentum transfer, separating charged reaction partners from the neutron spectators in momentum space (Fig. 1).

**Fig. 2:**
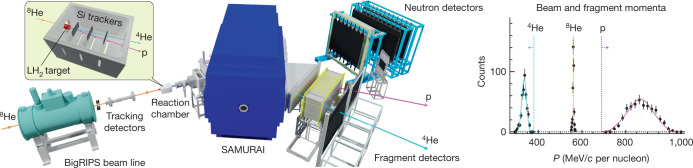
Experimental set-up and charged fragments momenta. Left: schematic view of the experimental set-up. The ^8^He secondary beam at 156 MeV per nucleon is transported from the BigRIPS (Big RIKEN projectile-fragment separator) into the SAMURAI set-up, where it hits a liquid-hydrogen (LH_2_) target. In a quasi-elastic $({\rm{p}},\,{{\rm{p}}}^{4}{\rm{He}})$ reaction, the ^4^He core is knocked out from the ^8^He projectile. Scintillator detectors and drift chambers are used for beam identification and tracking. The trajectories of the outgoing fragments are tracked by three silicon (Si) planes and bent afterwards through the SAMURAI spectrometer towards the focal-plane detectors. Two neutron-detector arrays were placed at a forward angle behind the SAMURAI. An additional scintillator wall was placed at smaller bending angle to detect the unreacted ^8^He beam. Right: measured momenta of the knocked-out ^4^He and the scattered proton after the quasi-elastic scattering (symbols). The momentum distribution of the incoming ^8^He beam is shown for comparison. The solid curves are the results from the simulation. The cyan (magenta) dotted line represents the upper (lower) limit of the ^4^He (proton) momentum expected from the simulation assuming a quasi-elastic scattering, and the orange line indicates the central beam momentum. Source data

The experiment took place at the Radioactive Ion Beam Factory operated by the RIKEN Nishina Center and the Center for Nuclear Study, University of Tokyo, using the Superconducting Analyzer for Multi-particles from Radio Isotope Beams (SAMURAI)^14^. A primary beam of ^18^O was directed onto a beryllium production target producing a cocktail of radioactive nuclei from fragmentation. The secondary ^8^He beam was separated using the BigRIPS fragment separator and transported with an energy of 156 MeV per nucleon to a 5-cm-thick liquid-hydrogen target^15^ located at the SAMURAI spectrometer (Fig. 2).

**Extended Data Fig. 1:**
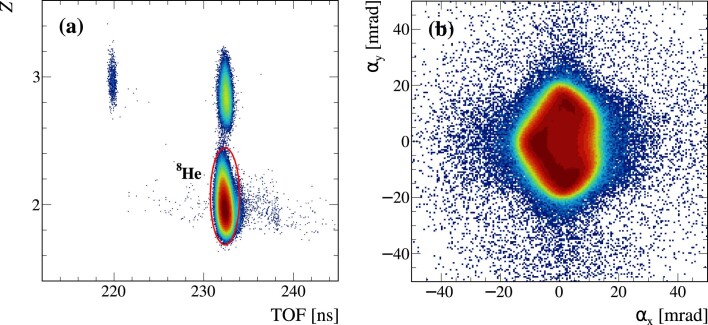
Incoming beam identification and tracking. (**a**) Charge identification vs. time-of-flight (TOF) for incoming beam ions measured event-by-event using plastic scintillators. The TOF is measured relative to the last scintillators at the BigRIPS fragment separator. The red ellipse represents the cut used to identify ^8^He ions. The peak above ^8^He, with the same TOF is attributed to pile-up events. The peak at smaller TOF and *Z* = 3 originates from Li. (**b**) Angular profile of the incoming beam in the XY plane measured by two drift chambers Source data

The incoming beam was measured upstream of the target on an event-by-event basis using scintillators for charge identification as well as momentum measurement, and two drift chambers for tracking (Extended Data Fig. 1).

**Extended Data Fig. 2:**
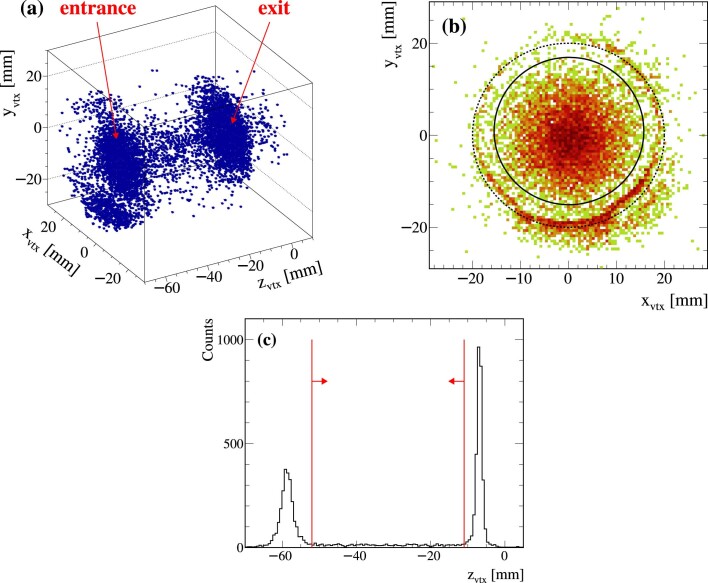
Reaction vertex. (**a**) 3D representation of the reconstructed reaction vertex from empty target runs. In such a case the incoming beam particle can interact with the material of the target cell at the entrance/exit of the cell, or with the residual hydrogen gas inside the target cell. (**b**) Reconstructed vertex in the XY plane. The region outside the solid circle is excluded in the analysis, to avoid reactions produced at the target cell and the support structure of the target. The dashed circle indicates the target cell, which has radius of 20 mm. (**c**) Projection onto the *z*–axis. The red lines represent the cut applied in the analysis. By fitting the peaks with Gaussians distributions, the vertex resolution is extracted to be ${\boldsymbol{\sigma }}=1.69\pm 0.04\,{\rm{mm}}$ at the entrance of the target, and ${\boldsymbol{\sigma }}=0.71\pm 0.01\,{\rm{mm}}$ at the exit of the target. The first silicon plane in these plots is located at ***z*** = 0 mm Source data

**Extended Data Fig. 3:**
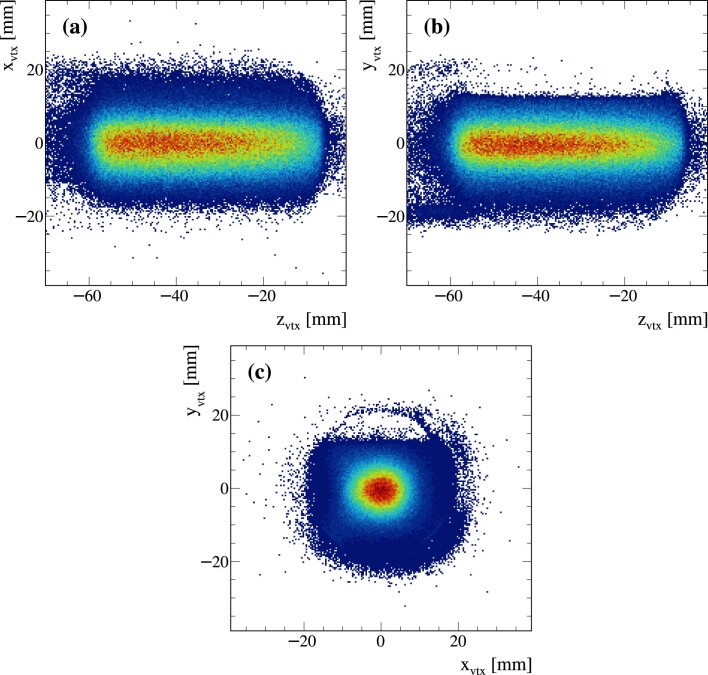
Target reconstruction. Reconstructed reaction vertex with the liquid-hydrogen target in ZX (a), ZY (b), and XY (c) planes. The cylindrical shape of the target is visible with a thickness of ~ 50 mm, where the first silicon plane in these plots is located at *z* =  0 In *x*- and *y*-directions, the vertices are Gaussian distributed with a cutoff according to the target cell radius. In y–direction a lower upper cutoff is visible, indicating that the target cell was not completely filled by liquid-hydrogen Source data

The outgoing charged fragments (α-particle and proton) emerging from the quasi-elastic scattering were detected using a combination of detectors downstream of the target. Three planes of silicon-strip detectors, where each plane consists of two orthogonal layers enabling position measurements in both horizontal and vertical directions, served for tracking, energy-deposition measurement and reconstruction of the reaction vertex inside the target (Extended Data Figs. 2 and 3).

**Extended Data Fig. 4:**
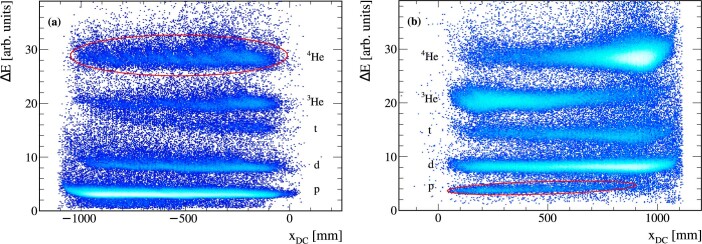
Charged fragments identification. Aligned energy deposition at each of the scintillator walls vs. the measured position in the drift chamber for events with two fragments. Different bands correspond to different fragments which are labelled. The left (right) scintillator wall (viewing opposite to the beam direction in Fig. 2) covers fragments emerging with relatively small (large) momenta. The red ellipses represent the cuts used to identify ‘slow‘ ^4^He and ‘fast‘ protons (see Supplementary Information) Source data

Behind the silicon planes, both charged fragments were bent through the magnetic field of the SAMURAI spectrometer, which was operated at a nominal magnetic field of 1.25 T in the centre of the magnet. The experiment was designed to detect an α-particle and a proton that emerge from quasi-elastic scattering close to 180° in the centre-of-mass frame (Fig. 1). Under these kinematical conditions, their resulting outgoing momenta are very different from each other in the laboratory frame, as shown in Figs. 1 and 2. The knocked-out α-particle is slowed down from its initial momentum, that is, with the incoming beam velocity, to a momentum of about 330 MeV/*c* per nucleon after the reaction (where *c* is the speed of light). In contrast, the proton, which was at rest in the initial state, becomes the fastest particle in the reaction, gaining a typical momentum of about 860 MeV/*c*. At the focal plane, a drift chamber is used for tracking of the fragments after the magnet, and two scintillator walls located side by side, which cover a wide momentum range, are used for energy-deposition and time-of-flight measurements. The α-particle and proton are identified from a combination of their measured energy deposition, each in a different scintillator wall, and their position in the drift chamber (Extended Data Fig. 4). Their momenta are determined precisely from their reconstructed trajectories through the SAMURAI spectrometer.

As no additional momentum is transferred to the neutrons in the reaction, they continue moving with nearly beam velocity and can be detected, in principle, by the neutron detectors placed at a forward angle behind the SAMURAI spectrometer. The detection efficiency for neutrons is much lower than that for charged particles and decreases quickly as a function of the number of detected neutrons. The small p–^4^He elastic cross-section at backwards centre-of-mass angles of less than 1 microbarn (ref. ^16^) resulted in the relatively low statistics of 422 events obtained for the ${}^{8}{\rm{H}}{\rm{e}}({\rm{p}},\,{{\rm{p}}}^{4}{\rm{H}}{\rm{e}})$ reaction. These factors made it impossible to detect more than two neutrons in coincidence with the charged particles. Therefore, the neutron detection is not a part of the current study, aside from a consistency check (provided in Supplementary Information) of the near recoil-less production of the free neutrons.

The combined selection of event-by-event identification of incoming ^8^He-beam particles in coincidence with the knocked-out α-particle and the scattered proton defines the ${}^{8}{\rm{H}}{\rm{e}}({\rm{p}},\,{{\rm{p}}}^{4}{\rm{H}}{\rm{e}})$ channel. From a precise measurement of the momenta of the charged particles, the energy spectrum of the 4n system is reconstructed assuming energy and momentum conservation through the missing mass:

1 $${E}_{4{\rm{n}}}=\sqrt{{E}_{{\rm{miss}}}^{2}-{{\bf{P}}}_{{\rm{miss}}}^{2}}-4{m}_{{\rm{n}}},$$

where *E*_miss_ (**P**_miss_) is the energy (momentum) component of the missing-momentum four-vector, and *m*_n_ is the neutron mass. Using this notation, a bound ^4^n system will appear at *E*_4n_ < 0 whereas a resonant state will appear at *E*_4n_ > 0. The missing momentum in equation (1) is defined by ${\bar{P}}_{{\rm{miss}}}={\bar{P}}_{{}^{8}{\rm{He}}}+{\bar{P}}_{{\rm{p}}({\rm{tgt}})}-{\bar{P}}_{{}^{4}{\rm{He}}}-{\bar{P}}_{{\rm{p}}}$, where the four-momenta $\bar{P}$ on the right-hand side of the equation are those of the incoming beam, target proton, knocked-out α-particle and scattered proton, respectively.

**Fig. 3:**
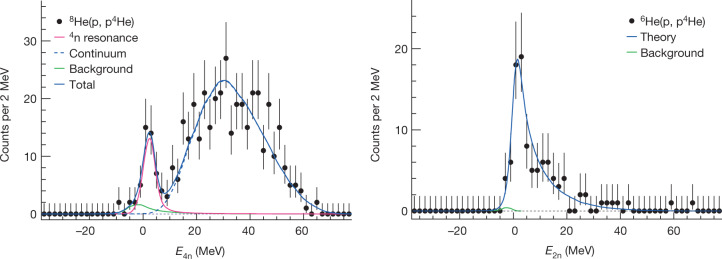
Missing-mass spectra. Left: missing-mass spectrum of the four-neutron system extracted from the ${}^{8}{\rm{He}}({\rm{p}},\,{{\rm{p}}}^{4}{\rm{He}})$ reaction. The different curves represent a Breit–Wigner resonance (pink), a non-resonant continuum (dashed blue), the background from two-step processes (green) and the total sum (solid blue). Right: missing-mass spectrum of the two-neutron system extracted from the ${}^{6}{\rm{He}}({\rm{p}},\,{{\rm{p}}}^{4}{\rm{He}})$ reaction. The blue curve represents the theoretical calculation^17^ convoluted with the experimental acceptance and resolution, and the green curve represents the background from the two-step reaction. Source data

The ${}^{6}{\rm{H}}{\rm{e}}({\rm{p}},{{\rm{p}}}^{4}{\rm{H}}{\rm{e}})$ knockout reaction was measured with almost exactly the same experimental conditions as for ^8^He, except for some small differences in the energy of the incoming beam and the beam profile (Supplementary Table 2), and served as a benchmark for verifying the analysis and calibration procedures. In the case of ^6^He, the 2n system is produced by the sudden removal of the ^4^He core. The two-neutron relative-energy spectrum is expected to be well described by theory taking into account both the well established ground-state wavefunction and the final-state scattering wave of the two neutrons, predicting a low-energy peak around 100 keV. Similarly to the ^8^He case, we define the missing mass (${\bar{P}}_{{}^{8}{\rm{He}}}\to {\bar{P}}_{{}^{6}{\rm{He}}}$ and $4{m}_{{\rm{n}}}\to 2{m}_{{\rm{n}}}$).The measured missing-mass spectrum for ^6^He is shown in the right panel of Fig. 3 together with the theoretical calculation^17^ convoluted with the experimental acceptance and resolution (blue curve). The energy range shown represents the one covered by the experimental acceptance. The calculation is compared with the data by implementing it into an event generator for the quasi-elastic reaction, which uses the measured p–^4^He differential elastic cross-section^16^ as an input, as well as the measured internal momentum distribution of the α-particle in ^6^He (ref. ^18^). The generated events are transported through the experimental set-up in Geant4 simulations to account for the experimental acceptance and detector resolutions. The excellent agreement of the simulated theoretical distribution with the measured spectrum confirms the analysis and the calibration for determining the missing mass. The missing-mass resolution obtained in the measurement is approximately 1 MeV sigma, and is almost constant over the measured energy range. The systematic uncertainty for the determination of the absolute energy was estimated from this measurement to be 0.4 MeV and that of the energy width to be 0.27 MeV (Methods). Also shown in the right panel of Fig. 3 (green curve) is a possible small background contribution coming from two-step process where ^4^He is produced in a first step (see Methods and following discussion for ^8^He). This background was estimated from the measured cross-section to contribute 1% of the total number of measured events.

The measured missing-mass spectrum of the four-neutron system from the ${}^{8}{\rm{He}}({\rm{p}},\,{{\rm{p}}}^{4}{\rm{H}}{\rm{e}})$ reaction is shown in the left panel of Fig. 3. Two components are observed: a well pronounced peak in the low-energy region with an energy around 2 MeV and a broad distribution at higher energies attributed to a non-resonant continuum response^13^, a direct four-body decay.

The shape of the non-resonant continuum spectrum of the four neutrons has been studied theoretically for the case of the four-neutron structure formed after the sudden removal of the α-core from ^8^He (ref. ^13^). The creation of the system is investigated by introducing into the Schrödinger equation a source term that accounts for the reaction mechanism producing the four-body system, and that depends explicitly on the internal structure of the parent nucleus ^8^He. The ^8^He ground-state wavefunction (without final-sate interaction) was treated using the five-body (${}^{4}{\rm{He}}+4{\rm{n}}$) cluster orbital shell model approximation (COSMA)^12^. The exact shape of the non-resonant continuum is sensitive to the hyperradius of the source, *ρ*_sour_ an internal radius of the 4n system, described in the hyperspherical harmonics basis. A hyperradius of 5.6 fm is considered by the theory as the most realistic, as it reproduces the correct experimental radius of ^8^He in the COSMA model. This results in a broad distribution centred around 30 MeV, in good agreement with the observed experimental spectrum.

We model the spectrum as follows:

2 $$f({E}_{4{\rm{n}}})=a{f}_{{\rm{res}}}({E}_{4{\rm{n}}})+b{f}_{{\rm{con}}}({E}_{4{\rm{n}}})+c{f}_{{\rm{bkg}}}({E}_{4{\rm{n}}}),$$

where *a*, *b*, and *c* are constants, *f*_res_ is a Breit–Wigner function representing the possible resonance structure, and *f*_con_ is the non-resonant continuum part presented above with the hyperradius as a parameter. The last term in equation (2), *f*_bkg_, represents possible background events coming from competing processes. Several processes were investigated and quantified (Methods), where the only non-negligible contribution found is from a two-step process involving ^6^He (^4^He) production: proton-induced break-up of ^8^He into ^6^He (^4^He) followed by a p–^4^He quasi-elastic scattering. The resulting energy distribution is broadened and shifted to lower energies compared with the pure ^6^He case (right panel of Fig. 3) owing to the two-step process, which has been taken into account in the simulation of *f*_bkg_. This background was estimated from measured cross-sections to contribute 2.6% to the total number of measured events (Methods), which has been used to determine the normalization constant *c*.

**Extended Data Fig. 5:**
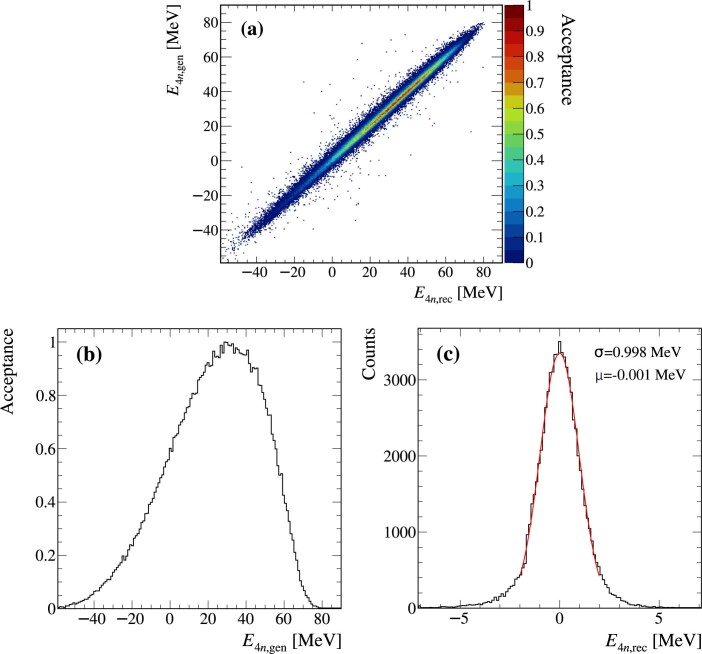
Energy response of the set-up. (a) Generated vs. reconstructed energies of the four-neutron system for a uniformly distributed generated energy in the range $-60 < {{\rm{E}}}_{4{\boldsymbol{n}},{\rm{gen}}} < 100\,{\rm{MeV}},$ transported through the experimental set-up. A clear diagonal correlation between the generated and reconstructed energies is observed. The colour code represents the acceptance of the set-up, normalized such that the maximum corresponds to one. (b) Projection of the acceptance as a function of the generated energy. (c) Projection of the response, as an example, for generated energy of zero MeV. The fitted width of the distribution of 1 MeV corresponds to the resolution of the reconstructed energy Source data

The experimental spectrum was then fitted with the energy-dependent function given in equation (2), where the fit function was convoluted with the experimental response, taking into account acceptance and detector resolutions. The experimental acceptance is not constant over the measured energy range. It is maximal in the region $20\,{\rm{MeV}} < {E}_{4{\rm{n}}} < 40\,{\rm{MeV}}$ (Extended Data Fig. 5).

The result of the *χ*^2^ minimization is presented by the solid blue curve in the left panel of Fig. 3, together with the individual contributions. The statistical significance of the peak structure is well beyond the 5*σ* level (Methods).

The probability of populating a four-neutron system in a resonant state after the sudden removal of the α-core in ^8^He is determined by the overlap of the 4n wavefunction in the final state and the relative motion of the four neutrons in the ^8^He initial state. This overlap integral defines the ratio of cross-sections for the population of the resonance and the non-resonant continuum. Unconvoluting with the acceptance of the set-up, following the energy dependence of equation (2), we extract a probability of *P*_r_ = 18.7 ± 2.3%. For comparison, the relative motion of the four neutrons studied in the COSMA model^12,13^ yields a probability of about 30%. This value is obtained by considering the hyperradius of 5.6 fm, whereas the resulting value from the fit to the experimental data is 5.0 ± 0.1 fm, which would yield a smaller probability to populate the resonant state.

Assuming a resonant state, its energy and width as determined from the fit are

$$\begin{array}{c}{E}_{{\rm{r}}}=2.37\pm 0.38({\rm{stat}}.)\pm 0.44({\rm{sys.}})\,{\rm{MeV}},\\ \varGamma =1.75\pm 0.22({\rm{stat}}.)\pm 0.30({\rm{sys.}})\,{\rm{MeV}}.\end{array}$$

**Fig. 4:**
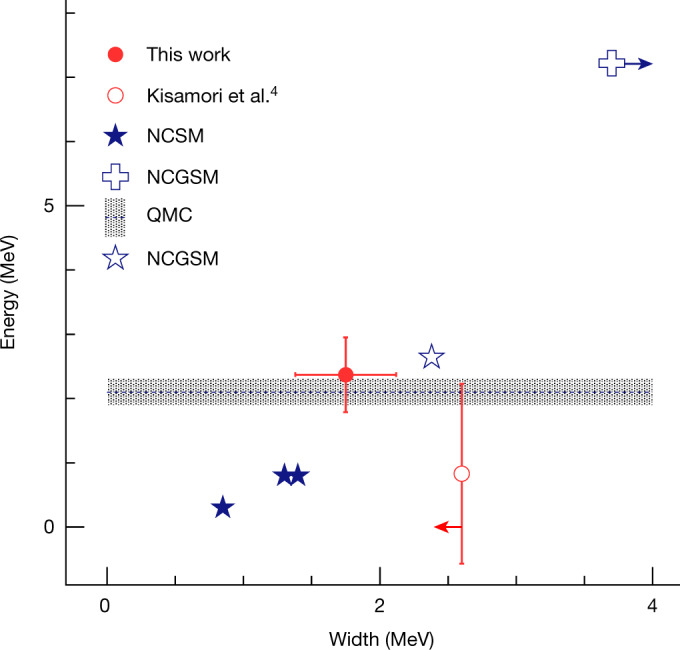
Comparison of experimental results with theory predictions. Energy versus width of a tetraneutron resonance. Experimental data are shown in red: this work (full symbol), and the result from the DCX measurement^4^ (open symbol), where the red arrow indicates that the measured width is an upper limit. Theory predictions are shown in blue based on: NCSM^19^ and ref. 20 cited in ref. ^20^ (full stars), NCGSM (open star)^20^ (cross)^21^, where the blue arrow indicates that the width is predicted to be larger than 3.7 MeV, and QMC calculations^5^ (band). Whether this observation of a low-energy peak is attributed to a four-neutron resonant state or to other correlations between the neutrons in the final state, needs to be clarified by ab initio theories. Source data

For comparison, Fig. 4 shows our result (full red symbol) together with the previous experimental result obtained from the DCX measurement^4^ (open red symbol). The energy of the resonance is in agreement within the uncertainty, despite the fact that different reactions were used to probe the 4n system, and is also in agreement with the upper limit given in ref. ^3^.

From the theory side, there is no consensus among the different theories and their predictions are partly contradictory, although, there is a general agreement that a bound ^4^n does not exist. In 2003, Pieper^10^ studied this possibility using Green’s function Monte Carlo calculations. His conclusion was that the existence of a bound ^4^n state has to be excluded, unless nuclear forces are drastically modified. However, his calculation suggested that a possible resonance might exist near 2 MeV, but in such a case it must be very broad.

Using a similar approach, the quantum Monte Carlo (QMC) framework based on two-body and three-body chiral interactions was used to calculate the energy of a ^4^n resonance^5^. The result supports the existence of a resonant state with an energy of 2.1(2) MeV, while no prediction has been made for its width (blue band). An extended no-core shell model (NCSM) approach using a harmonic-oscillator representation predicts different resonant states (full stars) including their corresponding widths^19^ (see also ref. ^20^ cited in ref. ^20^). Calculations have been performed also in the framework of the no-core Gamow shell model (NCGSM)^21^. These resulted in *E*_r_ ≈ 7 MeV and *Γ* ≈ 3.7 MeV (cross), where the conclusion was in fact that the energy of a ^4^n resonance might be compatible with the experimental value of ref. ^4^, albeit with a significantly larger width. As pointed out in a later study^20^, these calculations were incomplete, as they were performed only in truncated model spaces or with unphysically overbinding interactions. The authors of this work^20^ concluded that both the energy and the width of such a resonance are comparable with the experimental data (open star). At the same time, other calculations claim that to generate such a resonance, nuclear forces have to be significantly changed^22–27^, which would not be consistent with the present understanding. We note that some theories^26,27^ predict a non-resonant low-energy enhancement of the density of states in the four-neutron spectrum. Whether such a prediction is consistent with our observed resonance-like feature cannot be currently ascertained, as the energy spectrum of the four-neutron system is not given. The drastically different predictions resulting from different theoretical approaches highlight the importance of the current firm experimental observation.

In conclusion, we have presented the experimental observation of a resonance-like structure consistent with a tetraneutron state near threshold after 60 years of experimental attempts to clarify the existence of this state. The use of a high-energy knockout reaction in inverse kinematics allowed a precise measurement. The use of a radioactive ^8^He beam as the parent system and a direct, large momentum-transfer reaction opened up the opportunity to create the 4n system in a well defined one-step process and in a recoil-less undisturbed way. The optimized detection system enabled a precise determination of the final state and a high-resolution measurement. A well developed peak structure has been observed at an energy of 2.37 ± 0.38(stat.) ± 0.44(sys.) MeV with a striking statistical level. This is in agreement with the result of ref. ^4^ and the upper limit given in ref. ^3^. Both the energy and the extracted width of *Γ* = 1.75 ± 0.22(stat.) ± 0.30(sys.) MeV are consistent with a tetraneutron state that is unbound with a corresponding lifetime of (3.8 ± 0.8) × 10^−22^ s. Next-generation experiments using different reaction mechanisms and possibly detecting the four neutrons in coincidence will reveal more insights into the properties of the four-neutron system, including correlations among the neutrons. More elaborated ab initio nuclear theories accounting fully for the effect of the continuum are necessary to understand the observed low-energy peak and its origin.

## Methods

### Energy *E*_2n_ of the 2n system

**Extended Data Fig. 6:**
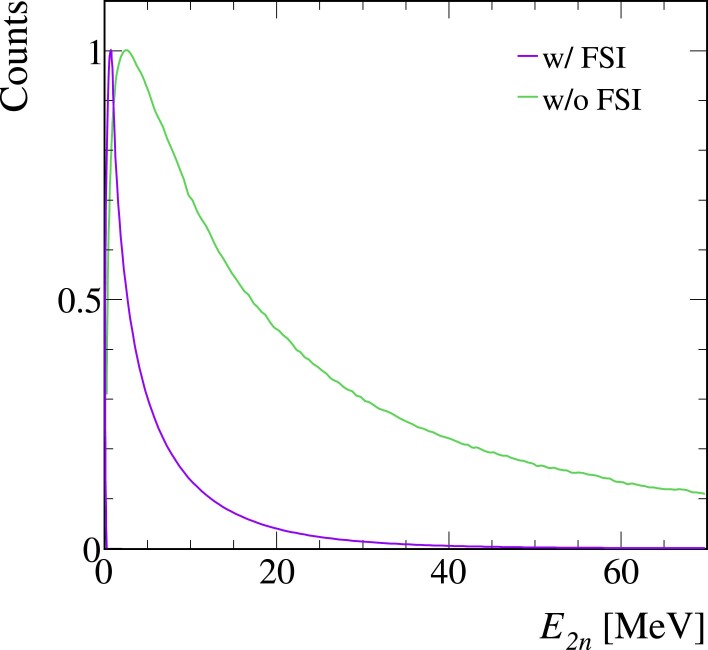
Energy of the 2n system. Theoretical *E*_2n_ distribution after ^4^He knockout from ^6^He resulting from the ^6^He ground-state wavefunction with (purple) and without (green) taking into account *nn* final-state interaction (FSI). Distributions are normalized such that their maximum is the same Source data

The theoretical calculation used as an input for the *E*_2n_ distribution includes both the ground-state wavefunction and the final-state interaction between the two neutrons^17^. These were obtained from a three-body (${}^{4}{\rm{He}}+2{\rm{n}}$) cluster model, which contains a phenomenological three-body force as well as the following local angular momentum *l-*dependent two-body interactions: a central *s*-wave interaction in the nn system, and central *s*- *p*- and *d*-wave interactions in the n–α system, as well as n–α spin–orbit interactions on the *p*- and *d*-waves. The ground-state wavefunction is computed in a three-body model using the computer code FaCE^28^, an acronym for Faddeev with Core Excitation, whereas the final-state interaction is taken into account by using the nn transition *t*-matrix approach. The calculated distribution is shown in Extended Data Fig. 6 (purple). For comparison, we also show the calculated distribution without the nn final-state interaction (green), which reflects the nn motion in the source, the ^6^He ground state. The distributions are normalized to the same maximum, such that only their shape is compared. It can be seen that the effect of the nn final-state interaction is very large. The ground-state distribution peaks at a larger energy with a far extending tail towards higher energies.

### Systematic uncertainty of the missing-mass determination

**Extended Data Fig. 7:**
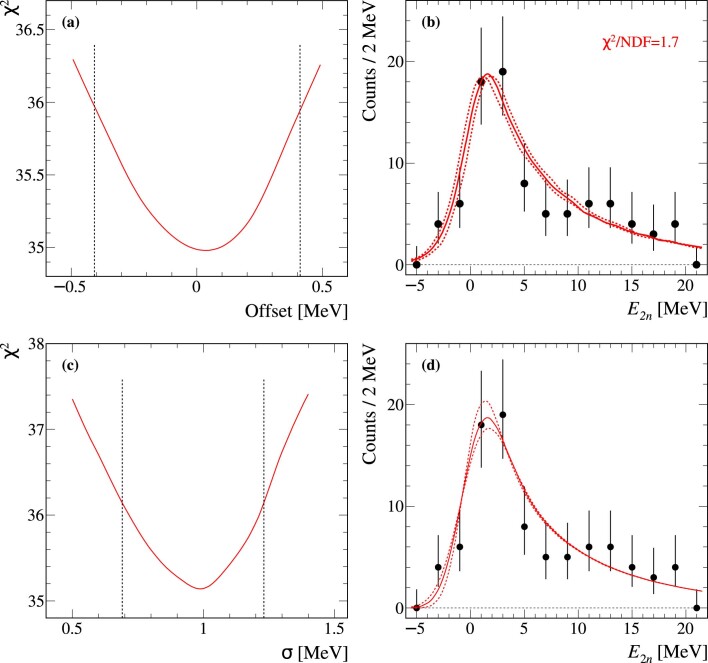
Estimation of the systematic uncertainty. (a) Calculated *χ*^2^ values as a function of the offset added to the theoretical distribution used to describe the energy of the two-neutron system in the ${}^{6}{\rm{H}}{\rm{e}}({\rm{p}},{\rm{p}}{}^{4}{\rm{H}}{\rm{e}})$ reaction. The systematic uncertainty for the energy is taken as the offset values at ${\chi }_{{\rm{\min }}}^{2}\pm 1,$ shown by the dashed lines. (b) The corresponding distributions of the data (black symbols), the original (solid red) and the shifted (dashed red) theoretical curves. Distributions are presented for the low-energy region, where the difference between the theory curves is visible. (c) Same as (a) but as a function of the energy resolution (using an offset of zero). The systematic uncertainty for the width is taken as the resolution values at ${\chi }_{{\rm{\min }}}^{2}\pm 1,$ shown by the dashed lines. (d) The corresponding distributions of the data (black symbols), the original (solid red) and the ${\chi }_{{\rm{\min }}}^{2}\pm 1$ resolution smeared (dashed red) theoretical curves. For comparison, the original distribution is also shown, where only the acceptance of the set-up has been taken into account but not the resolution, *i.e*., a resolution of zero MeV has been assumed. The distribution is scaled by a factor of 1/2 to match the scale of the figure Source data

As the theoretical calculation used as an input for the energy of the two-neutron system in the quasi-elastic ${}^{6}{\rm{He}}({\rm{p}},\,{\rm{p}}{}^{4}{\rm{He}})$ reaction is considered to be accurate, the comparison of it with the measured spectrum is used to estimate the systematic uncertainty of the missing-mass measurement. Different offsets covering the range of ±0.5 MeV are added to the generated theoretical distribution and for each offset the goodness of the agreement with the data is quantified by calculating the *χ*^2^ value. The generated distributions are convoluted with the energy response of the set-up, to account for the experimental acceptance and the detector resolutions. The minimum *χ*^2^ obtained is located very close to zero, that is, very close to the original distribution (Extended Data Fig. 7). The range of deviations in the energy scale still showing reasonable agreement with the data (${\chi }_{{\rm{\min }}}^{2}\pm 1$ range) is interpreted as the systematic uncertainty for the absolute missing-mass determination. This results in an uncertainty with an average value of ±0.4 MeV. To determine the systematic uncertainty for the missing-mass width, the original theoretical distribution is used (that is, offset of zero). To check the sensitivity to the resolution, different resolutions are applied in the range $\sigma =1\pm 0.5\,{\rm{MeV}}\,.$ The generated distributions are convoluted with the acceptance of the set-up and are smeared using a Gaussian function with a width *σ*. Similarly as described above, the *χ*^2^ values are calculated and the ${\chi }_{{\rm{\min }}}^{2}\pm 1$ range is interpreted as the systematic uncertainty (Extended Data Fig. 7). This results in an uncertainty with an average value of ±0.27 MeV. We note that for both absolute value and resolution, the minimum *χ*^2^ values fall very close to the expected values (which could be statistically by accident). As the given systematic uncertainties are determined solely by the statistics of the ^6^He data, we consider the given uncertainties as conservative estimates.

An additional systematic uncertainty from the background function in equation (2), on both the energy and the width, is added quadratically. The fitting procedure is repeated three times, with the green curve presented in Fig. 3 and with the 1*σ* lower and upper limits (Supplementary Information). The values for the energy and the width are taken as the average between the different fits, and the systematic uncertainty is taken as the difference between them. This results in an uncertainty of 0.18 MeV and 0.14 MeV for the energy and width, respectively.

### Energy *E*_4n_ of the 4n system

The relative-energy spectrum of four neutrons produced by sudden removal of the α-particle from ^8^He as a source was studied theoretically in ref. ^13^. The final-state energy spectrum depends on the intrinsic 4n relative motion in ^8^He, that is, the ^8^He ground-state wavefunction as the source (without final-state interaction), and the final scattering state of the four isolated neutrons, that is, 4n final-state interaction. The wavefunction used in ref. ^13^ for ^8^He is based on the COSMA model^12^, and the source term contains the Fourier transform of the overlap between ^8^He and the α-particle wavefunctions. The method of hyperspherical harmonics^29^ was used to solve the equations of the model. This method is based on the link between the hyperspherical-function method and the oscillator NCSM and uses a Slater determinant representation of the hyperspherical harmonics functions. The resulting source function is described by the internal variables of the four-neutron system, hyperradius, hyperangle, hyperangular momentum and the ^4^He–4n relative motion. Calculations showed that only hyperangular momentum *K* = 2 significantly populates the 4n non-resonant continuum, reflecting the motion of the four neutrons in ^8^He. The resulting energy spectrum in ref. ^13^ is presented only up to 20 MeV. It was also used in ref. ^4^ to estimate the non-resonant background and can be modelled analytically as

3 $${f}_{{\rm{cont}}}({E}_{4{\rm{n}}})={E}_{4{\rm{n}}}^{\alpha }\times \exp (-{E}_{4{\rm{n}}}/{\epsilon }_{{\rm{a}}}),$$

where *α* = 7/2 + *K* with *K* = 2 as stated above. *K* = 0 and *α* = 7/2 corresponds to the free four-body phase space. ${\epsilon }_{{\rm{a}}}\approx \frac{3.3{\hbar }^{2}}{{m}_{{\rm{N}}}{\rho }_{{\rm{sour}}}^{2}},$ where *m*_N_ is the nucleon mass, *ρ*_sour_ is the hyperradius, *ħ* is the reduced Planck constant and the factor 3.3 was estimated by matching to the calculated distribution in ref. ^13^.

### Background events from competing reactions

Events with a proton and an α-particle in the final state might result from other competing reactions. However, owing to the unique kinematics of quasi-elastic p–^4^He scattering at large centre-of-mass angles, other direct reactions are excluded. Examples are one-neutron knockout, ${}^{8}{\rm{He}}{({\rm{p}},{\rm{p}}{\rm{n}})}^{7}{\rm{He}},$ or ^6^He knockout, ${}^{8}{\rm{He}}({\rm{p}},\,{{\rm{p}}}^{6}{{\rm{He}}}^{* })2{\rm{n}}.$ In the former case, the momentum of the proton is too small to be selected by the experiment, where in the latter, it is too high at the angular range covered by the set-up, owing to the larger mass of ^6^He relative to ^4^He Therefore, the only possible contribution of background events to our measured p–^4^He events can come from secondary reactions. Below we list the possible reactions and their expected contributions.

${}^{8}{\rm{He}}{({\rm{p}},{\rm{pn}})}^{7}{\rm{He}}$. A first interaction is single-neutron knockout from ^8^He leading to break-up into ^6^He and two neutrons. This can be followed by a second interaction, quasi-elastic p–^4^He scattering at backward centre-of-mass angles. These processes will result in events with a missing-mass distribution similar to that of the one-step ${}^{6}{\rm{He}}({\rm{p}},\,{{\rm{p}}}^{4}{\rm{He}})$ reaction but broadened and shifted to smaller energies. This is due to the difference in the separation energies of two and four neutrons in ^6^He versus ^8^He of about 2 MeV, and due to the additional recoil (Supplementary Information). The single-neutron knockout cross-sections from ^6,8^He were measured in an experiment performed within the same experimental campaign as the experiment presented here, at a slightly higher beam energy of 185 MeV per nucleon using a series of targets with different nuclear charges *Z* (ref. ^30^). For ^6^He, the cross-section was extracted for the hydrogen target, whereas for ^8^He, the lowest*-Z* target used was carbon. We therefore scale the measured cross-section for ^6^He with hydrogen, ${\sigma }_{{}^{6}{\rm{He}},{\rm{H}}}$, by the carbon measurement $({\sigma }_{{}^{8}{\rm{He}},{\rm{C}}}/{\sigma }_{{}^{6}{\rm{He}},{\rm{C}}})=1.26\pm 0.16.$. Using ${\sigma }_{{}^{6}{\rm{He}},{\rm{H}}}=47\pm 4\,{\rm{mb}}$ results in ${\sigma }_{1{\rm{n}}}=59\pm 9\,{\rm{mb}}$.

${}^{8}{\rm{He}}{({\rm{p}},{\rm{p}})}^{8}{{\rm{He}}}^{* }$. A first interaction is inelastic excitation of ^8^He leading to a break-up into ^6^He and two neutrons. These cross-sections were measured as well in ref. ^30^, with scaling ratio of ${\sigma }_{{}^{8}{\rm{He}},{\rm{C}}}/{\sigma }_{{}^{6}{\rm{He}},{\rm{C}}}=1.02\pm 0.13.$ Using ${\sigma }_{{}^{6}{\rm{He}},{\rm{H}}}=11.0\pm 0.9\,{\rm{mb}}$ gives ${\sigma }_{{\rm{inel}}}=11.2\pm 1.7\,{\rm{mb}}$.

In total, the cross-section evaluated for the two processes described above equals $${\sigma }_{{\rm{tot}}}=71.1\pm 9.2\,{\rm{mb}}\,.$$ Evaluating the relative contribution to the number of one-step p–^4^He quasi-elastic reactions *N*_reac_ at half of the target thickness (*t*) gives $${N}_{{\rm{bkg}}}/{N}_{{\rm{reac}}}\,=\sigma ({\rm{b}})\times t({\rm{g}}\,{{\rm{cm}}}^{-2})\times $$ $$0.6({\rm{Avogadro}})/{\rm{A}}=0.071\times 0.175\times 0.6$$$$=\,0.75\pm 0.10 \% $$, where A is the mass number of the target. These types of processes can also occur from ^8^He break-up along beamline materials before the target, plastic scintillators, kapton windows and air. These contributions were estimated starting from the last plastic scintillators at the BigRIPS fragment separator up to the kapton window at the entrance to the target cell, using the measured cross-sections for hydrogen and carbon targets^30^. The produced ^6^He has an angular spread in the transverse direction according to its intrinsic momentum, which has a width of $${\sigma }_{{}^{6}{\rm{He}}}=35\,{\rm{MeV}}/c$$ (ref. ^18^). Assuming a central beam energy of 156 AMeV, its corresponding momentum is $${P}_{{}^{6}{\rm{He}}}=3.37\,{\rm{GeV}}/c$$, leading to an angular *θ* width of $${\sigma }_{\theta }={\sigma }_{{}^{6}{\rm{He}}}/{P}_{{}^{6}{\rm{He}}}=0.035/3.37=10\,{\rm{mrad}}.$$ The spread in the *x* and *y* directions evaluated at the entrance to the target equals $${\sigma }_{x,y}=d\cdot {\sigma }_{\theta }=d({\rm{mm}})\times 0.01({\rm{rad}})\,,$$ where *d* is the distance from the material at which ^6^He was produced to the target entrance. The target enclosure radius equals 20 mm. Therefore, depending on the distance of production point and target, some fraction of the produced ^6^He is expected to not reach the target, which reduces its intensity. Using the measured distances to the target entrance, the estimated fraction of ^6^He produced before the target and reaching the effective target volume is 0.36 ± 0.01%. Overall, the estimated contribution from these two processes is 1.11 ± 0.10%.

${}^{8}{\rm{He}}({\rm{p}},\,{{\rm{p}}}^{6}{\rm{He}})2{\rm{n}}$. A first interaction is ^6^He knockout to its ground state. This process can contribute only when the ^6^He is produced along the target, as, owing to its angular spread, it will not reach the target region in case of production in beamline materials. The cross-section for this process is not well known, and was studied previously only for a much higher energy^18^. However, it was shown to be compatible with the p–^4^He elastic scattering cross-section. We therefore adapt the measured p–^4^He total cross-section, which was measured at 156 MeV (ref. ^16^), *σ* = 91.8 mb, and use it as an upper limit. The relative contribution of these events is evaluated as $0.0918\times 0.175\times 0.6=0.96 \%$.

${}^{8}{\rm{He}}+{\rm{p}}{\to }^{4}{\rm{He}}$ . In the first interaction, ^4^He is produced from fragmentation of ^8^He. This can be, for example, a result of elastic p–^4^He scattering or inelastic excitation of ^8^He. The inclusive ^4^He cross-section was measured at higher energy for ${}^{8}{\rm{He}}+{}^{12}{\rm{C}}\to {}^{4}{\rm{He}}$ to be 95 ± 5 mb (ref. ^31^). We scale it by a factor of 1/2 to estimate the cross-section for the hydrogen target, leading to *σ* = 48 ± 5 mb. The relative contribution of these events is evaluated as $0.048\times 0.175\times 0.6=0.50\pm 0.05 \% .$ This process will result in events with a negative missing-mass distribution below −3.1 MeV, the binding energy of ^4^He in ^8^He that will extend to more negative values owing to the additional recoil (Supplementary Information).

${}^{8}{\rm{He}}{({\rm{p}},{\rm{p}})}^{8}{\rm{He}}$. A first interaction is p–^8^He elastic scattering at backwards centre-of-mass angles. This will lead to a fast proton in the final state. In a second interaction, ^4^He can be produced. However, in this case, the momentum of the proton is even larger than that resulting from direct ^6^He knockout, owing to the larger mass; therefore, this reaction channel is excluded.

For the benchmark measurement with ^6^He, only one reaction can contribute to a background, where ^4^He is produced in the first interaction, ${}^{6}{\rm{He}}+{\rm{p}}{\to }^{4}{\rm{He}}$. Similar to ^8^He, the inclusive ^4^He cross-section was measured at higher energy for ${}^{6}{\rm{He}}+{}^{12}{\rm{C}}\to {}^{4}{\rm{He}}$ to be 189 ± 14 mb (ref. ^31^). We scale it by a factor of 1/2 to estimate the cross-section for the hydrogen target, leading to *σ* = 95 ± 7 mb. The relative contribution of these events is evaluated as $0.095\times 0.175\times 0.6=1.00\pm 0.07 \% .$ This process will result in events with a negative missing-mass distribution below −0.975 MeV, the binding energy of ^4^He in ^6^He that will extend to more negative values owing to the additional recoil (Supplementary Information).

### Summary of background contributions

From the reactions listed above, we conclude that the only contributions from background processes come from reactions in which ^6^He or ^4^He were produced in a first step, and the overall contribution is evaluated as 2.6%. In the fitting of the missing-mass spectrum, *f*_bgk_ is taken as the simulated distribution for the two-step processes described above. Each process is simulated individually, and adds weight to the total distribution according to its expected contribution. In addition, as the measured cross-sections adopted to estimate the background contributions were not measured directly for the reactions of interest, we consider the uncertainty as a variation by a factor of two on each one of the scaled cross-sections (Supplementary Information).

It is noted that the resulting background distribution can also explain the two events observed at the energy region of $-10\,{\rm{MeV}} < {E}_{4{\rm{n}}} < -\,8\,{\rm{MeV}}.$ These two events cannot result from a direct reaction, as the lower limit of a bound tetraneutron is −3.1 MeV, corresponding to the binding energy of ^8^He against decay into ${}^{4}{\rm{He}}+4{\rm{n}}$. Therefore, these events can be attributed only to two-step reactions. Estimating the background function at −9 MeV, we expect 0.27 events in that energy region, leading to a 2*σ* significance of the measured two events, which is not considered as a statistically significant deviation from the expected background contribution in that energy region.

**Extended Data Fig. 8:**
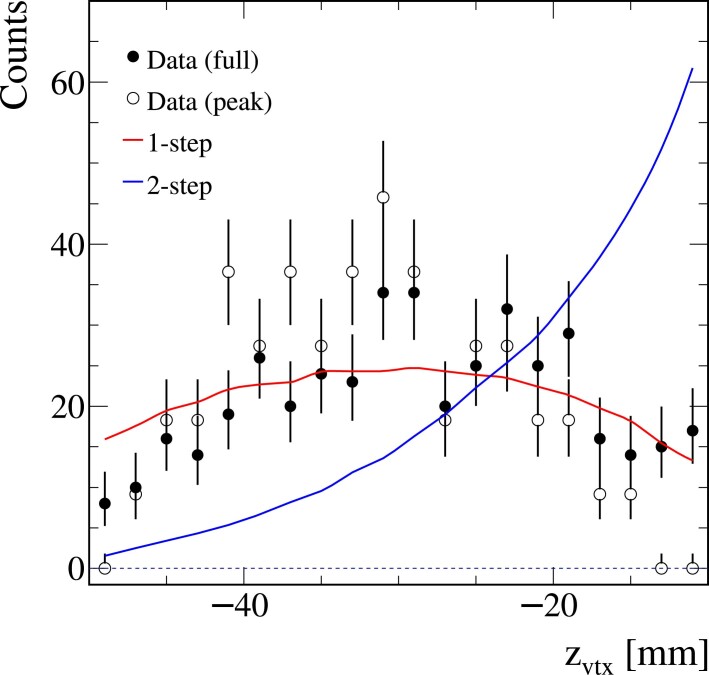
Reaction vertex of the selected events. Reaction vertex for the selected quasi-elastic ${}^{8}{\rm{H}}{\rm{e}}({\rm{p}},{\rm{p}}{}^{4}{\rm{H}}{\rm{e}})$ events. Data sample is shown by the full symbols, and the low-energy peak region is shown by the open symbols and normalized according to the number of events for the full data set, together with a simulation of one-step process (red curve), and two-step process (blue curve): break-up of ^8^He into ^6^He followed by quasi-elastic ${\rm{p}}-{}^{4}{\rm{H}}{\rm{e}}$ scattering. As can be seen, an exponential increase is expected towards the exit of the target, which is not observed in the data, confirming that the contribution from this background is indeed as small as expected from the evaluation based on measured cross-sections. Simulated distributions for other two-step processes show the same exponential behaviour Source data

Finally, a major difference between one-step and two-step processes is expected in the reaction-vertex distribution of the proton and the α-particle (Extended Data Fig. 8). For a two-step process, an exponentially increasing yield of reaction products along the target is expected owing to the need to produce the ^6^He (^4^He) first. Such a distribution is not observed in the data, corroborating that the background contribution is indeed small. The estimated background contribution will be considered to evaluate the significance level of the observed peak.

To quantify the statistical significance of the resonance observed in Fig. 3, we evaluate the number of background events in the peak region defined as ${E}_{{\rm{r}}}\pm 2\varGamma$ and $-2\,{\rm{MeV}} < {E}_{4{\rm{n}}} < 6\,{\rm{MeV}}.$ The number of measured events in the peak region amounts to *N* = 41 events. The number of background events equals the integral of the background and continuum functions (*f*_bkg_ and *f*_cont_ in equation (2)) in that region, resulting in an average number of eight events from the background and two events from the continuum, such that the total expected background amounts to ten events. Even though we allowed a variation of a factor of two for the background estimation, this does not change the result significantly. Hence, we conclude that the resonance structure is observed with high statistical significance, well above the 5*σ* observation threshold.

## Online content

Any methods, additional references, Nature Research reporting summaries, source data, extended data, supplementary information, acknowledgements, peer review information; details of author contributions and competing interests; and statements of data and code availability are available at 10.1038/s41586-022-04827-6.

## Supplementary information

Supplementary Information This Supplementary Information file contains the following sections. Section 1, SAMURAI set-up; Section 2, Beam measurement; Section 3, Fragments measurement; Section 4, Quasi-elastic events. It includes Supplementary Figs, 1–29, Tables 1–4 and references.

## Data Availability

The datasets generated during and/or analysed during the current study are available from the corresponding author on reasonable request. Source data are provided with this paper.

## Source data

Source Data Fig. 2
Source Data Fig. 3
Source Data Fig. 4
Source Data Extended Data Fig. 1
Source Data Extended Data Fig. 2
Source Data Extended Data Fig. 3
Source Data Extended Data Fig. 4
Source Data Extended Data Fig. 5
Source Data Extended Data Fig. 6
Source Data Extended Data Fig. 7
Source Data Extended Data Fig. 8
