# Supplementary material for: Observation of a correlated free four-neutron system
Source: Nature. 2022 Jun 22;606(7915):678–82. doi: 10.1038/s41586-022-04827-6 (PMC9217746; doi:10.1038/s41586-022-04827-6)
Supplement: Supplementary file 1 — This Supplementary Information file contains the following sections. Section 1, SAMURAI set-up; Section 2, Beam measurement; Section 3, Fragments measurement; Section 4, Quasi-elastic events. It includes Supplementary Figs, 1–29, Tables 1–4 and references. [file 41586_2022_4827_MOESM1_ESM.pdf]

nature portfolio

https://doi.org/10.1038/s41586-022-04827-6

Supplementary information

Observation of a correlated free four-neutron system

In the format provided by the authors and unedited

Supplementary Materials

1 SAMURAI setup

The SAMURAI experimental setup used in the experiment was configured such that the outgoing alpha and proton particles emerging from quasi-elastic scattering at large momentum transfer corresponding to $160^\circ < \theta_{\text{c.m.}} < 180^\circ$ in the center-of-mass (c.m.) frame, can be measured in coincidence with high resolution through the large acceptance superconducting dipole magnet. The setup is shown in Fig. 1 of the main text and described in the following. Technical details on the SAMURAI spectrometer can be found in [1].

- **Scintillators for Beam TOF (SBTs)** - The first detectors installed at the entrance to the SAMURAI area are two plastic-scintillators, SBT1 and SBT2 with a thickness of 2 mm. They are used to measure time and energy deposition, from which the TOF and nuclear charge Z, of the incoming beam are extracted. Each scintillator is equipped with two PMTs that are read by TDC and QDC for time and energy conversion, respectively. The TOF is measured relative to the last two plastic scintillators at the BigRIPS fragment separator.
- **Beam Drift Chambers (BDCs)** - To determine the angular profile of the incoming beam, two drift chambers are used. They have an effective area of 8×8 cm^2, with a maximum drift-length of 2.5 mm. Each BDC consists of four double layers structured as XX-YY-XX-YY, where Y layers are rotated by 90°, for tracking in both, vertical and horizontal directions. The wires in each layer are read out by a TDC, to measure the drift time.
- **Liquid-hydrogen target** - In order to maximize the number of proton-induced knock-out reactions, and keeping at the same time the angular- and energy-straggling as low as possible, a liquid-hydrogen target with a density of 0.07 g/cm^3 was chosen. A 5 cm MINOS (Magic Number Off Stability) target [2] was used in the experiment, with a 120 μm thick mylar enclosure and radius of 2 cm.
- **Silicon Trackers** - The silicon trackers are the first detectors placed after the target and are used for tracking of the charged particles before the SAMURAI magnet, energy deposition measurement, and reconstruction of the reaction vertex along the target. The particles are tracked by their signals in the strips, which give the hit position as well as their deposited energy. The detectors concept is based on single-sided silicon strip detectors with a thickness and pitch of 100 μm. It consists of three identical planes separated by 12 cm from each other, each has an effective area of 8×5 cm^2. Every plane is composed of both X and Y layers to provide spatial position measurement in both directions, where the two layers are separated by 4.8 mm. The vertex reconstruction resolution depends on the distance of the first silicon plane from the target. To achieve a good resolution, it should be placed as close as possible to the target. A safe distance of 6 mm between the first silicon plane and the exit window of the target was chosen.

  The detectors were contained in a vacuum chamber with dimensions $49 \times 36 \times 27$ cm^3 and a wall thickness of 25 mm, that was connected to the target chamber vacuum-tight.
- **SAMURAI Magnet** - The SAMURAI magnet is a large acceptance superconducting dipole magnet used for bending of the charged particles, such that their momenta are determined by their measured trajectories through the magnet. The magnet was positioned in its standard configuration of 30° with respect to the beam axis. It was operated during the experiment with a nominal magnetic field of 1.25 T.
- **Forward Drift Chamber (FDC2)** - At the focal-plane after the SAMURAI magnet, a drift chamber, FDC2, is used for tracking of charged particles. It has an effective area of 229.6×83.6 cm^2, with a maximum drift length of 10 mm. It consists of seven double layers structured as XX-UU-VV-XX-UU-VV-XX, where U and V layers are rotated by $\pm 30^\circ$, respectively. The detector was positioned at 60° relative to the beam axis.
- **Hodoscopes (HODs)** - A hodoscope is an array of plastic scintillators used for energy deposition and TOF measurement (the TOF is relative to the start time defined by SBTs) of charged particles. This, in combination with signals from other detectors allows to perform the fragment identification. Two hodoscopes were placed after FDC2, positioned at 60° relative to the beam axis: hodoscope for fragments (HODF), consists of 24 bars, and hodoscope for protons (HODP) consists of 16 bars, out of which 14 were operated. Figure 1 shows the HODs alignment as seen by the beam. A gap between the two HODs was filled by two overlapping bars from

the veto wall of the neutron detector NEBULA, labeled as bars 24, 26. The overlap between the two bars is labeled as 'bar 25' even though it is not in itself a separate physical plastic bar. Each bar of HODF, HODP has a size $120 \times 10 \times 1$ cm^3. Similarly to SBTs, each bar is equipped with two PMTs. An additional hodoscope, HODS, consists of only seven bars was placed in 22° relative to the beam axis, used to detect unreacted ^{8}He beam particles.

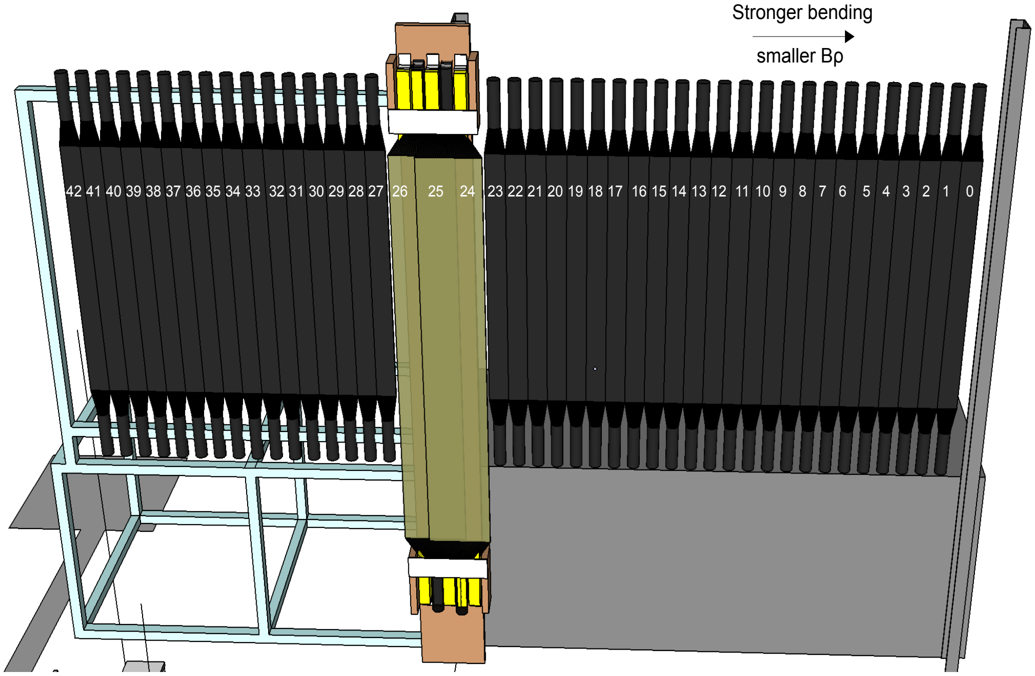

Supplementary Fig. 1: Schematics of the HODs alignment in the experiment as seen by the beam. Bar 25 represents the overlap region between the two wider bars (24 and 26) that were used to cover the gap between the two HODs.

- **NeuLAND and NEBULA** - Two neutron detectors were placed in forward angle after the SAMURAI magnet. The NeuLAND detector concept is based on double-planes, where each double-plane consists of 50 horizontally and 50 vertically scintillator bars. Each bar has a size of 5×5 cm^2 and 250 cm length. In total four such double-planes were installed. The NEBULA detector consists of 120 scintillator bars, all arranged vertically, with a size of 12×12 cm^2 and 180 cm length. The detector is arranged as two separated walls, each with 60 bars, divided into two layers. Additionally, 12 bars with dimension of $190 \times 32 \times 1$ cm^3 were placed before each NEBULA wall to veto background from charged particles. As stated in the main paper, neutron detection is out of the scope of the current analysis, and data from the neutron detectors is used only as a consistency check (see Sec. 4.5).

- **Data Acquisition (DAQ) and Triggers** - The DAQ system used in the experiment consists of three independent branches, one for the neutron detectors (NeuLAND and NEBULA), one for the silicon trackers, and one for the rest of the detectors. These DAQ branches were connected by the trigger. Six different triggers were used, listed in Table 1 and shown statistically in Fig. 2. Trigger (0) is the beam trigger: it requires only the incoming beam entering the SAMURAI area, and is triggered by a signal at the SBTs detectors. Other triggers include the beam trigger combined with additional detectors in coincidence. In these cases, the beam trigger is referred to as a signal of the incoming beam in anti-coincidence with HODS, i.e, no unreacted beam. Our main trigger of interest is trigger (5), as the alpha and proton are detected at HODF and HODP, respectively. Beam trigger (0) was downscaled by a factor of 1000, and the neutron triggers (1) and (2) were downscaled by a factor of 5.

Supplementary Table 1: Coincidence triggers used for data collection.

Trigger	BEAM	HODF	HODP	NEBULA	NeuLAND
0	x				
1	x			x	
2	x				x
3	x	x		x	
4	x			x	x
5	x	x	x		

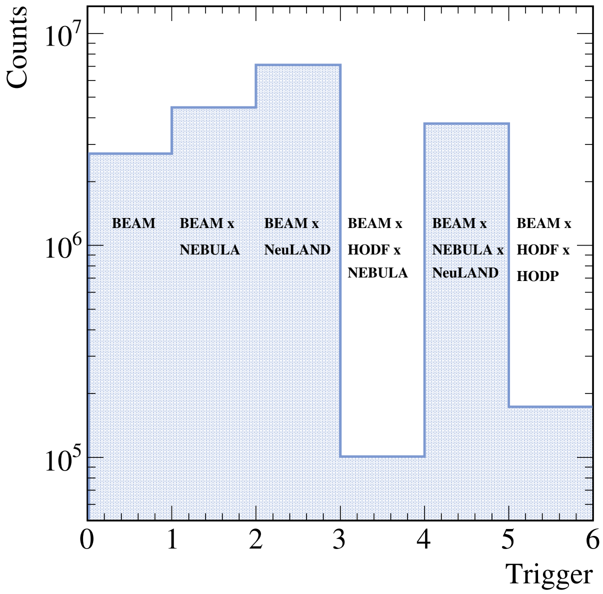

Supplementary Fig. 2: Statistics of the coincidence triggers used for data collection.

2 Beam measurement

The main data in the experiment were collected for a setting of ^{8}He beam with the liquid-hydrogen target. In the 1st data set of the ^{8}He runs, which accounts for about half of the data, time information from the HODs was not available due to a technical problem with the constant fraction discriminators. This was then fixed for the 2nd half of the ^{8}He runs (referred to as second ^{8}He data set). Additional difference between the two sets of runs came from a change along the BigRIPS fragment separator, at the first focal-plane (out of seven), labeled as F1, the width of the slits used for ions transportation was relatively wide, 30 mm, in the first set of ^{8}He runs, compared to 2.5 mm in the second set. Therefore, this led to a wider beam profile in the first data set. Lastly, the beam centering at the SAMURAI setup using the BDCs was not identical between the two data sets. In addition to ^{8}He settings, data were collected with ^{6}He beam used to verify the analysis and calibration procedures. These runs were taken in between the two sets of ^{8}He runs, where time information from the HODs was collected, while other conditions were similar to those of the 1st ^{8}He data set. For calibration of different detectors, several runs were taken with a proton beam and an empty target with various experimental conditions. Table 2 summarizes the characteristics of the different settings.

The beam identification is done using plastic scintillator detectors: two scintillators placed at the last focal-plane of the BigRIPS fragment separator, labeled as F7, and the two SBTs. From measurements of the energy deposition and the TOF between F7 and SBTs, the nuclear charge Z of the incoming ions is extracted. Figure 3 shows the incoming identification plot for the 1st ^{8}He (a) and ^{6}He (b) settings, whereas the 2nd ^{8}He setting is shown in Fig. 1 (a) of the

Supplementary Table 2: Characteristics of the different settings used in the experiment. Central Bρ values are given between the last focal-plane at the BigRIPS fragment separator and SBT. Rates for the different settings represent the averaged beam rate. Measurement time and rate for the proton beam setting are not shown here since different configurations were used for different calibrations purposes.

Setting	Target	F1 slits [mm]	Central Bρ [Tm]	Measurement time [h]	Rate [kHz]
1st ^{8}He	LH$_2$	30	7.5367	36.1	176
	Empty			2.8	
^{6}He	LH$_2$	30	5.6644	16.7	160
	Empty			2.5	
2nd ^{8}He	LH$_2$	2.5	7.5367	41.7	185
p	Empty	1	2.0032		

Extended Data. The cuts used for incoming identification are shown by the red boxes. The peaks above the ^{8,6}He peaks, with the same TOF and larger nuclear charge Z correspond to $Z = \sqrt{2} \cdot 2$, originate from pile-up events, a result of two ions arriving at the same time to SBT. Events at smaller TOF and $Z = 3$ originate from Li isotopes. For ^{8}He only a very small amount of incoming Li is observed, which reflects the high purity of the beam, whereas for ^{6}He the amount of $Z = 3$ ions is significantly larger, as the beam purity was worse for this less exotic nucleus.

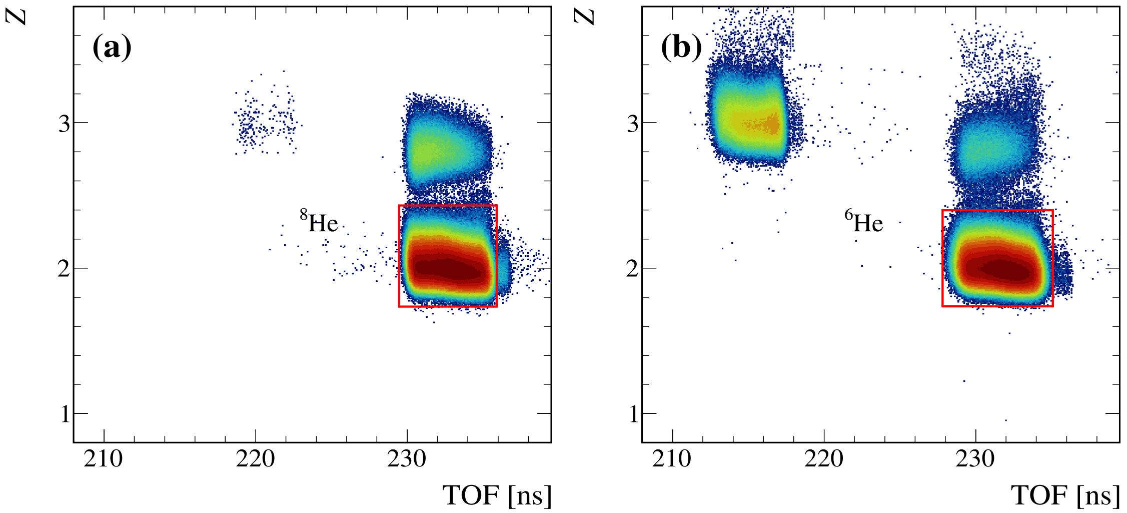

Supplementary Fig. 3: Incoming beam identification plot for (a) 1st set of ^{8}He, (b) ^{6}He. Cuts used for beam identification are shown by the red boxes.

Using the measured TOF the velocity of the incoming beam particle is calculated, and its kinetic energy is extracted. The TOF resolution is 84 ps sigma. Figure 4 shows the incoming kinetic energy for the different settings. Distributions are normalized to the same integral value such that only their shape is compared. The distributions are wider for the first ^{8}He and ^{6}He settings (red and black) due to the larger width of the slits at F1. In addition, the central energy for ^{6}He is larger than ^{8}He, see Bρ values listed in Table 2.

The kinetic energy shown in Fig 4 is that at the entrance to SBT. To evaluate the incoming energy at the reaction vertex two energy loss corrections are applied. First, the energy loss of the incoming beam in beamline materials in considered, starting from SBT1 until the target entrance. This is evaluated using the SAMURAI simulator software, a Geant4 [3] based simulation at which the exact setup of the experiment was implemented. We extract the energy loss as a function of the incoming energy, which is then evaluated on an event-by-event basis. Since the liquid-hydrogen target used in the experiment was rather thick (5 cm), the second energy loss correction is that at the target according to

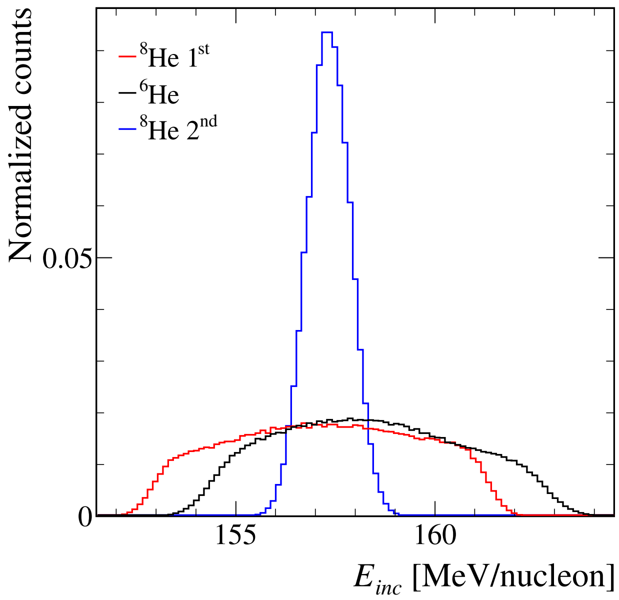

Supplementary Fig. 4: Incoming kinetic energy for the 1st set of ^{8}He (red), ^{6}He (black), and (c) 2nd set of ^{8}He (blue). Distributions are normalized to the same integral value such that only their shape is compared.

the reconstructed reaction vertex along the target. The vertex is reconstructed using the silicon trackers (see Sec. 3.1). The energy loss is calculated using ATIMA code [4] and applied to the kinetic energy on an event-by-event basis according to the reaction vertex.

The beam tracking is achieved using BDC1 and BDC2. First, the measured drift time is converted into a drift length using the TDC signals and a space-time conversion. In a second step, a track is reconstructed by combining the hits in different layers, where we request hits in all X and Y layers for each BDC. The resulted position resolution averaged over the different layers equals 152 μm sigma (151 μm) for BDC1 (BDC2). The angle of the incoming beam particle in x, y directions is obtained from the combined position measurement at the two BDCs. Figure 5 shows the angular profile for the 1st ^{8}He (a) and ^{6}He (b) settings, whereas the 2nd ^{8}He setting is shown in Fig. 1 (b) of the Extended Data. As stated above, centering of the beam on the BDCs was not the same for all settings. To ensure that the differences observed do not come from different alignment of the BDCs wires, the angular beam profile was compared with that measured by the silicon trackers, by considering events with unreacted beam. The exact same distributions were observed.

The detection efficiencies of the BDCs are extracted relative to the incoming beam particles that passed the incoming identification cut, defined by the plastic scintillators, resulted in an efficiency of 96% (94%) for BDC1 (BDC2).

3 Fragments measurement

3.1 Charged particles detectors

Since the main goal of the analysis was to measure in coincidence a fast proton and a slow alpha emerging from quasi-elastic scattering at backward c.m. angles, in the following we focus on the detection of two charged particles in the HODs, FDC2, and silicon trackers.

HODs For fragment identification the standard method is to use both the energy deposition and TOF measurements. As stated in Sec. 2, for about half of the ^{8}He data time information from the HODs is not available, therefore only the energy deposition is used. The time information that is available in the 2nd ^{8}He setting is used as a sanity check.

Figure 6 shows the energy deposition (left) in arbitrary units, and the measured time (right) as a function of the bar number for the second setting of ^{8}He. Shown are only bars that are covered by FDC2. The energy deposition of

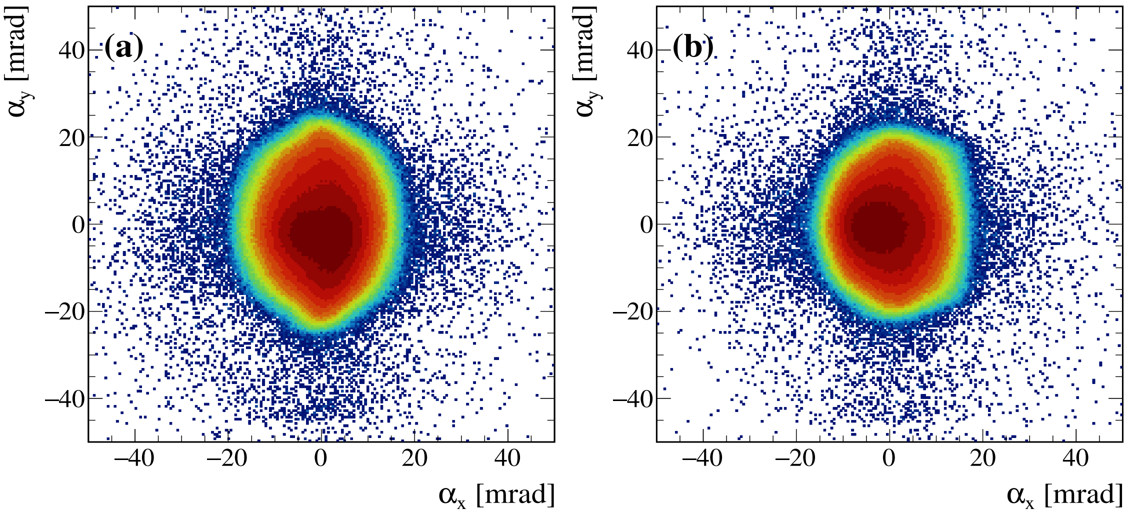

Supplementary Fig. 5: Angular profile of the incoming beam in the XY plane for (a) 1st set of ^{8}He, (b) ^{6}He as measured by the BDCs.

a certain fragment depends on its velocity, such that lower bar number at the HODs corresponds to smaller magnetic rigidity Bρ, as shown in Fig. 1. Therefore, fragments hitting HODF are relatively slow, and those hitting HODP are relatively fast, such that the alpha and proton from the reaction of interest are expected to hit HODF and HODP, respectively. The energy deposition shown in Fig. 6 (left) is aligned such that it has the same central value for a specific fragment. The different bands represent the energy deposited by different fragments. In an increasing order of energies, these are protons, deuterons, tritons, ^{3}He, and ^{4}He. At even higher energies, ^{6}He is also visible, which in general is expected to hit the region between HODP and HODS. The overlap between the two bars placed at the gap between HODF and HODP, bar 25, is considered in case both gap bars (24 and 26) fired.

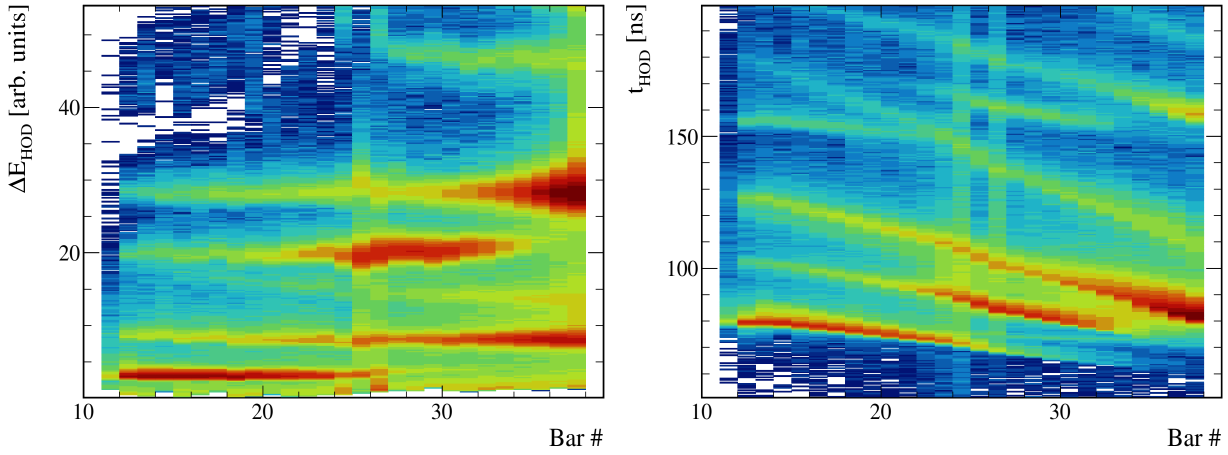

Supplementary Fig. 6: Aligned energy loss (left) and measured time (right) at the HODs as a function of the bar number for the 2nd ^{8}He setting. Shown only bars that are covered by FDC2. Different energy bands represent different fragments. In an increasing order of energies, these are protons, deuterons, tritons, ^{3}He, and ^{4}He.

To separate the alpha and proton well enough, the gap bars and an additional bar from each side of the gap, at the edges of the two HODs are excluded. In the following, whenever we refer to HODF this corresponds to bars 11-22, and HODP to 27-37.

The starting point for identifying two fragments is by requiring hit multiplicity-2 at the HODs, such that $M_{HODP} = 1$ and $M_{HODF} = 1$. Figure 7 shows the hit multiplicity at HODP vs. HODF, where the red numbers on top represent the fraction of events with a certain multiplicity combination in percentage.

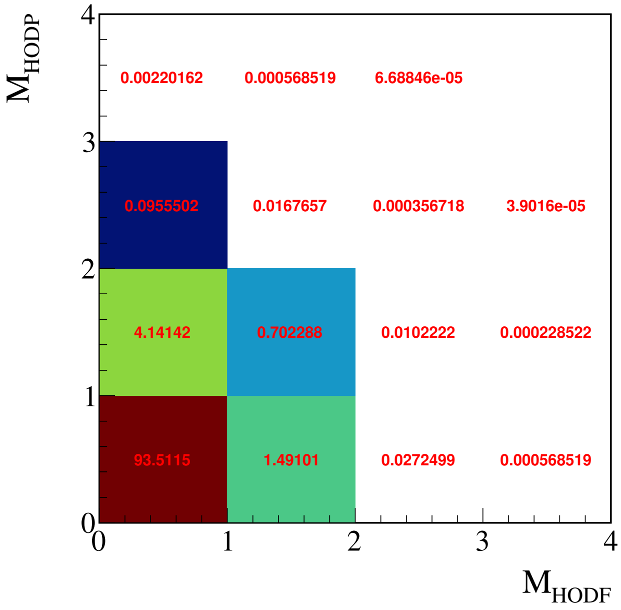

Supplementary Fig. 7: Hit multiplicity at HODP vs. HODF. The red numbers represent the percentage of events with a certain multiplicity combination.

FDC2 For the fragments tracking at FDC2 only the hits from the X layers (dispersive plane) are considered in order to increase the efficiency. Tracks are reconstructed in a similar way to the BDCs, where we focus on the case where exactly two tracks were reconstructed. Figure 8 (left) shows the measured angle vs. position for $N_{tracks} = 2$ and hit multiplicity-2 at the HODs. The resulted position and angular resolutions equal 360 μm and 0.54 mrad sigma, respectively.

Most of the events lay on the diagonal region as expected, and two separated regions are observed. The first, labeled as left track, corresponds to fragments that hit HODF. The second, labeled as right track, corresponds to fragments that hit HODP. The gap between the two regions represent the gap region between the two HODs that is excluded. The black lines in Fig. 8 define the cuts used to select two tracks. A small fraction of events lay outside the diagonal region in Fig. 8 (left). Charged particles bend through the SAMURAI magnet and continue then in a straight line. Using the measured position and angle of the two reconstructed tracks, their 'vertex' z-point can be reconstructed. For events where the two particles originated from reaction in the target region, this point should be located far away from FDC2 region. Figure 9 shows the reconstructed vertex point for events where one of the two tracks lay outside the diagonal region. Two peaks are observed in the figure, represent tracks originated from the region between the exit window of the SAMURAI magnet, which is located at distance of about 500 mm from the center of FDC2, and the center of FDC2, assumed here to be located at $z = 0$. An additional so called "window cut" is applied with $z < -2000$ mm. Figure 8 (right) shows the measured angle vs. position after applying the window cut, most of the off diagonal events are removed. Finally, a linear correlation should exist between the extrapolated position at the HODs and the fired bar. Figure 10 shows the correlation between the two for HODF (left) and HODP (right). Indeed, a correlation exists, however, at the edges of the HODs, especially for HODP, a non diagonal region is observed. These events are excluded as shown by the red lines. It should be noted that when requiring a coincidence with the silicon trackers the intensity of these bands is strongly reduced. In addition, when selecting our QE sample, i.e., alpha at HODF and proton at HODP, as shown in Fig. 4 of the Extended Data, this corresponds to $x_{HOD} \gtrsim -1300$ mm and $x_{HOD} \lesssim 1050$ mm, respectively. Therefore, any contamination coming from the possible continuation of these bands to the diagonal region is excluded.

The detection efficiency can be extracted directly for a single track, and only evaluated for two tracks. In the

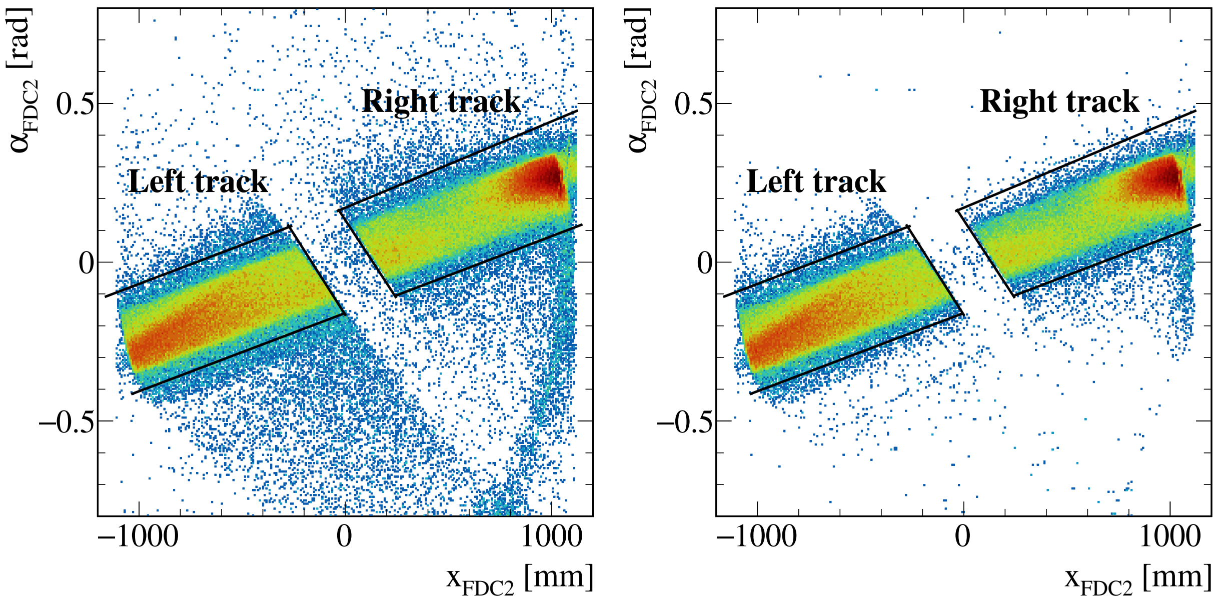

Supplementary Fig. 8: Left: Measured angle vs. position at FDC2 for events with $N_{tracks} = 2$ and hit multiplicity-2 at HODs, together with the cut used to define valid tracks (black lines). Right: Same but after applying the "window cut".

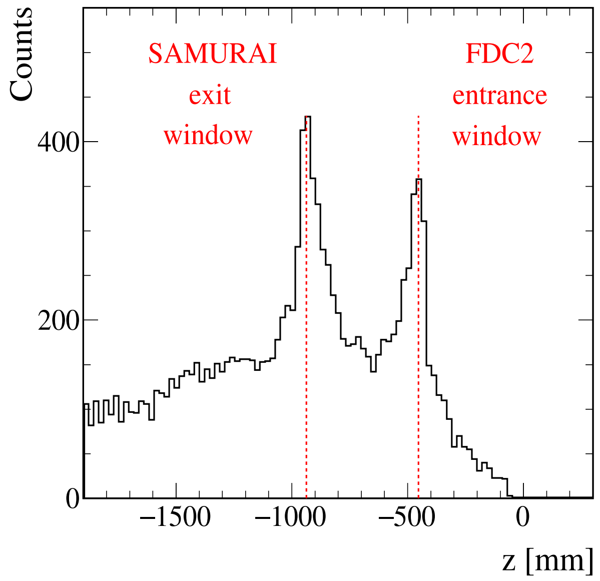

Supplementary Fig. 9: Reconstructed 'vertex' $z-$point from the measured angle and position of two tracks at FDC2, for events where one of the tracks lay in the off diagonal (outside the graphical cut) region shown in Fig. 8 (left). The peaks represent track pairs originated from the region between the exit window of the SAMURAI magnet and FDC2 entrance window. A cut of $z < -2000$ mm is applied to exclude these events.

case of a single track, proton calibration runs are used, as unreacted ^{6,8}He beam particles would have bent through the SAMURAI magnet only with relatively small angles which are not covered by FDC2. Two calibration runs with proton beam at energy of ~ 174 MeV were taken, using different magnetic fields, one with the nominal field of 1.25 T,

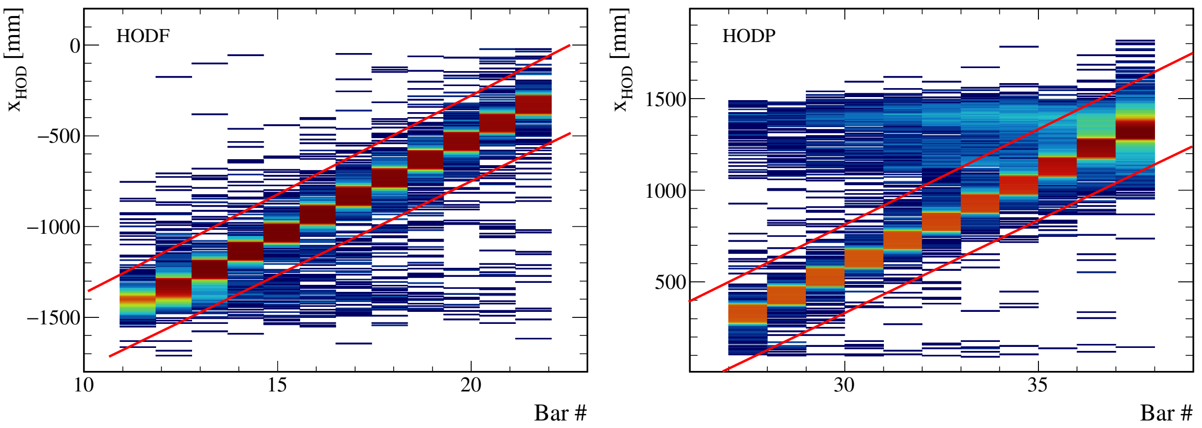

Supplementary Fig. 10: Correlation between the extrapolated position of the reconstructed tracks from FDC2 and the fired bar at HODF (left) and HODP (right).

and one with 0.8 T. These magnet settings correspond to protons hitting the left and right regions of FDC2 shown in Fig. 8, following then to HODF and HODP, respectively. We define the single track detection efficiency for protons as the ratio of events with a single reconstructed track at FDC2 relative to the number of incoming and outgoing protons

$$\varepsilon_{\mathrm{FDC2}} = \frac{(\text{Single track})\&(p_{\mathrm{in}})\&(p_{\mathrm{out}})}{(p_{\mathrm{in}})\&(p_{\mathrm{out}})}, \tag{1}$$

where p_{in} are the incoming protons identified in the same way as done for the incoming beam, and p_{out} are the outgoing protons measured by HODF or HODP for the different runs. An averaged efficiency of 96% was obtained. We assume that the efficiency is roughly the same for $Z = 2$ fragments. For the physics runs, with the liquid-hydrogen target, the exact value of the efficiency can not be extracted directly, since the fragment identification is not done by the HODs alone. As shown in Fig. 6 different bands should correspond to different fragments. To estimate the efficiency, we cut on a specific band and consider the fraction of events with a single reconstructed track. For ^{4}He we get a fraction of 91%, in a reasonable agreement with the extracted efficiency.

Similarly, in the case of two tracks, we estimate the fraction of events with two valid tracks at FDC2 relative to the HODs by cutting on two bands. Although the reaction of interest is a proton at HODP and ^{4}He at HODF, it can be seen from Fig. 6 that protons at HODP are not clearly visible, without including additional detectors. Therefore, for the efficiency estimation we consider the opposite case, which is clearly visible, *i.e.*, a proton at HODF and ^{4}He at HODP. The fraction of events with two valid tracks equals 67%, see Table 3 for details.

Note that protons at HODF are slower than those at HODP, such that they produce larger signals which lead to larger detection efficiency. Therefore, the efficiency estimation preformed above represents only an upper limit to the reaction of interest.

Silicon trackers In each one of the silicon layers the different hits are combined into clusters. A cluster is a group of neighboring fired strips in the same layer. The clusters are converted into energies by summing the neighboring strips and tracks are reconstructed in both X and Y directions. Our goal is to identify two tracks at the silicon trackers that originate from the same point, the reaction vertex. Due to the finite position resolution, the reconstructed tracks will not cross each other in a single point. We therefore use the approach of the closest point between two tracks. Figure 11 shows the minimum distance (MD) extracted for pairs of tracks. We apply a cut of MD<0.2 mm to ensure that the two tracks are coming from the same reaction. The vertex is then reconstructed, as shown in Figs. 2, 3 of the Extended Data, located at the center of the minimized distance vector, connecting the two tracks.

Similarly to FDC2 the detection efficiency can be extracted for a single track, and evaluated for two tracks. For the case of a single track, unlike FDC2, all the beam particles from the different empty target settings go through the trackers. Therefore, this allows to extract independently the detection efficiency for protons as well as ^{8}He, where the outgoing ^{8}He is identified at HODS. ^{6}He is excluded since it is expected to hit the region between HODP and HODS. The detection efficiency is defined similarly to Eq. 1, with an additional condition, that the measured energy deposition

Supplementary Table 3: The fraction of events with different track combinations at FDC2 relative to multiplicity-2 events at HODs with a rough cut on a proton and ^{4}He at HODF and HODP, respectively. Good left and right tracks refer to the two regions of FDC2 defined as shown in Fig. 8.

Tracks	%
2 HODs	100
0 tracks	5
1 track	17
2 tracks	77
2 + good left track	74
2 + good right track	73
2 + good left & right tracks	70
+ Window cut	69
+ HODs bar cut	67

of the reconstructed track matches the most probable energy loss value of the incoming particle within 2σ limits. This resulted in an efficiency of 93% and 94% for protons and ^{8}He, respectively. From the physics runs we estimate the efficiency for ^{4}He as 90%. For the case of two tracks, we estimate the fraction of the events with two valid tracks at the silicon trackers relative to the HODs. In the same manner done for FDC2, we consider the case of a proton at HODF and ^{4}He at HODP. The fraction of events with two tracks equals 49%, where the different contributions to this fraction are listed in Table 4.

Supplementary Table 4: The fraction of events with two tracks relative to multiplicity-2 events at HODs with a rough cut on a proton at HODF and ^{4}He at HODP. $N_{hits} > 1$ for a certain plane, means we require more than one cluster-hit in both X and Y layers of the plane. The vertex cut is shown in Fig. 2 of the Extended Data.

	%
2 HODs	100
$N_{hits} > 1$ 1st plane	67
$N_{hits} > 1$ 2nd plane	66
$N_{hits} > 1$ 3rd plane	65
$N_{hits} > 1$ all planes	60
2 tracks	57
MD cut	53
Vertex cut	49

3.2 Fragment identification and momentum

The standard method for fragment identification is the $\mathrm{TOF} - \mathrm{B}\rho - \Delta E$ method, which allows to extract both the nuclear charge Z and the mass-to-charge ratio A/Z. However, since time information is not available for about half of the ^{8}He data, we use an alternative way to identify the fragments, and then extract their $\mathrm{B}\rho$ for momentum measurement.

Figure 4 of the Extended Data shows the fragment identification plot at HODF (left) and HODP (right): the aligned energy deposition at the HODs vs. the measured position at FDC2, for events with multiplicity-2 at the HODs and two valid reconstructed tracks at FDC2. With the addition of FDC2, protons at HODP are now visible, and the red ellipses represent the cuts used to identify ^{4}He and proton emerging from quasi-elastic scattering at backward angles. It can be seen from the figure that the opposite case, *i.e.*, a proton at HODF and ^{4}He at HODP is the dominant one. This

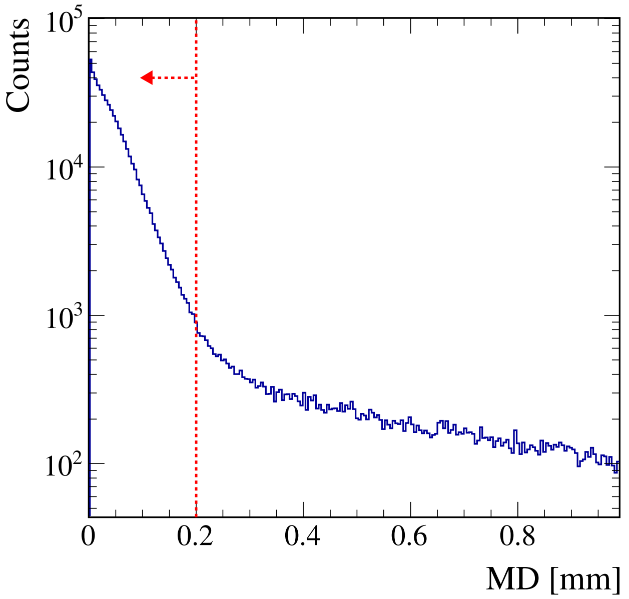

Supplementary Fig. 11: Vertex minimum distribution together with its cut.

corresponds to relatively slow protons and fast ^{4}He, compared to our quasi-elastic events, with momentum just above and below the central beam momentum, respectively. This is due to the setups acceptance, which do not cover proton and ^{4}He with momenta similar to the beam momentum. It should be noted that these events do not emerge from quasi-elastic scattering, which was confirmed in addition by a simulation, but from other processes such as for example, single-neutron knockout, ^{6}He knockout, or inelastic excitation from ^{8}He that will lead to an alpha that has roughly beam momentum, and the same for proton, as only protons from reaction at backward c.m. angles can be accepted by the setup. As the cross section for these processes is larger than that for quasi-elastic scattering at backward c.m. angles, ~ 1 mb compared to ~ 1 μb, the intensity is significantly larger.

To complete the identification we look at the measured energy deposition at the silicon trackers for both selected particles. The energy deposited in 100 μm silicon strip is very different for fast proton and slow alpha. For slow alphas, the expected energy deposition is more than 700 keV. For slow alpha particles which travel through the detector, the upper energy boundary of the detector squeezes all cases with an energy deposition higher than ~ 650 keV to the region of $\sim 500-600$ keV. For fast protons, the minimum deposited energy for the fastest protons is expected at ~ 60 keV. Figure 12 shows the energy deposition in the first X layer for the protons (red) and alphas (blue) from the selected coincident events. For protons it can be seen that the distribution is well centered at very low energy deposition as expected. For alphas the majority of the events are distributed around a central high energy deposition peak. A small tail is observed at lower energy deposition, attributed to faster alphas that were tagged. We exclude these events as shown by the right black arrow. Similarly, we apply a cut to exclude slower protons shown by the left black arrow. These cuts are applied on all six layers of the silicon trackers.

To check the fragment identification presented above, we use the ^{8}He data from the second half of the runs, which contains the time information from the HODs. In this case, the mass-to-charge ratio A/Z can be reconstructed from Bρ and TOF measurements. The Bρ measurement is described in the following. Figure 13 shows the A/Z ratios for the selected protons (left) and ^{4}He (right). The extracted ratios match the A/Z of one and two, respectively, confirming the fragment identification. A similar check was done with ^{6}He data, showing the same result.

Fragments are tracked before the SAMURAI magnet by the silicon trackers, bent through the magnet according to their magnetic rigidity, Bρ, and tracked again by FDC2. The magnetic rigidity can be expressed as

$$\mathrm{B}\rho = \frac{P}{Z}, \tag{2}$$

where P is the magnitude of the momentum of the fragment, and Z its nuclear charge. By measuring the deflection of an identified fragment, we extract its momentum. In combination with the direction vector measured by the silicon

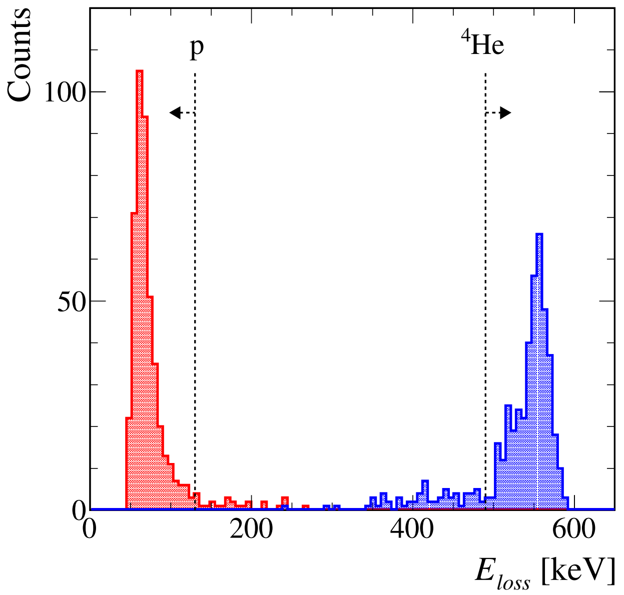

Supplementary Fig. 12: Measured energy deposition in the first X layer of the silicon trackers for events with fast protons (red) and slow ^{4}He (blue). To avoid contributions from fast ^{4}He and slow protons the cuts shown by the black lines are applied.

trackers, the momentum vector is determined. We derive a Bρ function using the Multi-Dimensional-Fit (MDF) [5] method with a simulated training sample. The simulated sample is created using the SAMURAI simulator software, a Geant4 [3] based simulation. The exact geometry of the setup is implemented, and the simulation is performed using a field map of the magnetic field used in the experiment. Fragment trajectories are generated with a large phase space to cover the full geometrical acceptance of the SAMURAI magnet. Similarly, the generated Bρ covers the full possible range accepted by the setup. We perform the simulation with an empty target setup, where the starting point of the fragment is just before the first silicon detector. To derive a Bρ function with the highest possible accuracy, for this purpose only, we vacuumed the different detectors, such that we do not take into account the multiple scattering of the fragments in the detectors. In this manner, we get a pure Bρ function, that will be naturally smeared by the experimental data. Using this approach the energy loss in the silicon trackers and in the liquid-hydrogen target are then corrected separately for protons and ^{4}He. With the training sample the MDF is then performed, Bρ is fitted as a function of six independent variables

$$\mathrm{B}\rho = f(\mathrm{x_{SI}}, \alpha_{\mathrm{x,SI}}, \mathrm{y_{SI}}, \alpha_{\mathrm{y,SI}}, \mathrm{x_{FDC2}}, \alpha_{\mathrm{FDC2}}), \tag{3}$$

where $\mathrm{x_{SI}}$ ($\mathrm{y_{SI}}$) is the measured x (y) position at the silicon tracker, $\alpha_{\mathrm{x,SI}}$ ($\alpha_{\mathrm{y,SI}}$) is the angle in the x (y) direction, and $\mathrm{x_{FDC2}}$ (α_{FDC2}) is the position (angle) at FDC2. The resulting function f is a sum of Monomials. The MDF procedure with the simulated training sample results in an analytical fit function, which can be applied to the measured positions and angles in the experimental data.

Figure 14 shows the MDF results for a training sample of ^{4}He fragments. The residual in the plots is defined as the relative difference between the reconstructed and generated Bρ, $(\mathrm{B}\rho_{\mathrm{mdf}} - \mathrm{B}\rho_{\mathrm{gen}})/\mathrm{B}\rho_{\mathrm{gen}}$. Figure 14 (left) shows the residual vs. $\mathrm{B}\rho_{\mathrm{gen}}$, which stays overall constant over the $\mathrm{B}\rho_{\mathrm{gen}}$ range. Figure 14 (right) shows the 1D projection of the residual. The resulting tracking precision obtained, without including detector resolutions is 0.06%.

To validate the MDF function we use the experimental data from the proton beam with empty target discussed in Sec. 3.1. For these runs the Bρ value at the entrance to the SAMURAI setup is known, and we correct for the energy loss in beamline materials using the Geant4 [3] simulation, and for the silicon detectors we calculated the energy loss in 100 μm of silicon using ATIMA code [4], and corrected the momentum iteratively, as there are six silicon layers. Using Eq. 2 this defines a known reference momentum, p_{ref}. Figure 15 shows the relative difference $(p_{\mathrm{mdf}} - p_{\mathrm{ref}})/p_{\mathrm{ref}}$, where p_{mdf} is the proton momentum evaluated for the measured positions using the MDF function.

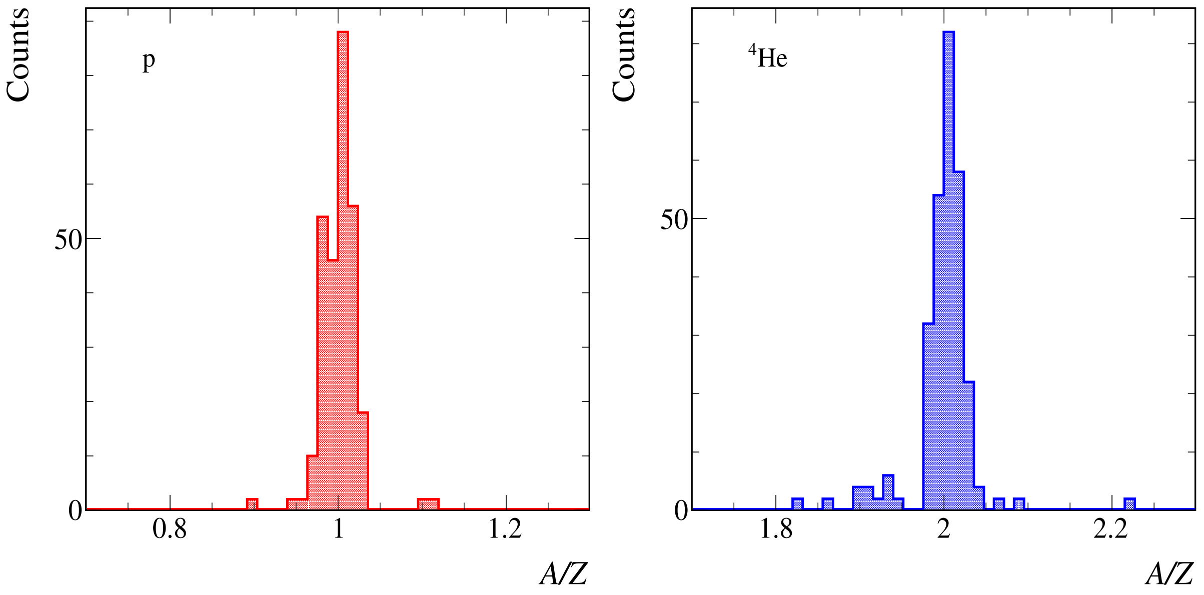

Supplementary Fig. 13: Extracted A/Z ratios for fast protons (left) and slow ^{4}He (right). Data shown for the 2nd set of ^{8}He runs, which includes time information from the HODs.

A relative momentum precision of 0.13% is obtained, which now includes the detector resolutions and the multiple scattering in the silicon detectors. In the physics runs the momentum resolution is further smeared due to the multiple scattering in the liquid-hydrogen target.

As presented above the MDF function was extracted for simulated data using empty target setup. Therefore, after evaluating the MDF function for the measured positions in the physics data, we correct for the energy loss of the proton and ^{4}He in the silicon detectors and the liquid-hydrogen target. For the silicon this is done in the same way as described above. For the liquid-hydrogen target, we correct the energy loss according to the reconstructed reaction vertex, where the energy loss is calculated using ATIMA code [4] and evaluated on an event-by-event basis.

4 Quasi-elastic events

The combined selection of identified event-by-event incoming ^{8}He ion in coincidence with a fast proton and slow alpha fragments defines the quasi-elastic $^8\text{He}(p, p^4\text{He})$ reaction at backward c.m. angles. The energy- and momentum conservation in the reaction relates the four-momenta of the involved particles as

$$\bar{P}_{^8\text{He}} + \bar{P}_{p(\text{tgt})} = \bar{P}_p + \bar{P}_{^4\text{He}} + \bar{P}_{4n}, \tag{4}$$

where the four-momenta represent the incoming beam, target proton, scattered proton, knocked-out alpha, and the total momentum of the four-neutron system, respectively. Using the measured four-momenta of the charged particles involved in the reaction, we can define the missing momentum vector

$$\bar{P}_{miss} = \bar{P}_{^8\text{He}} + \bar{P}_{p(\text{tgt})} - \bar{P}_p - \bar{P}_{^4\text{He}}, \tag{5}$$

which represents the total four-momentum of the $4n$ system. From the mass of the missing-momentum vector the relative energy of the four-neutron system is obtained

$$E_{4n} = \sqrt{E_{miss}^2 - \boldsymbol{P}_{miss}^2} - 4 \cdot m_n, \tag{6}$$

where m_n is the neutron mass. Reconstructing the E_{4n} spectrum reveals information about the correlations between the neutrons.

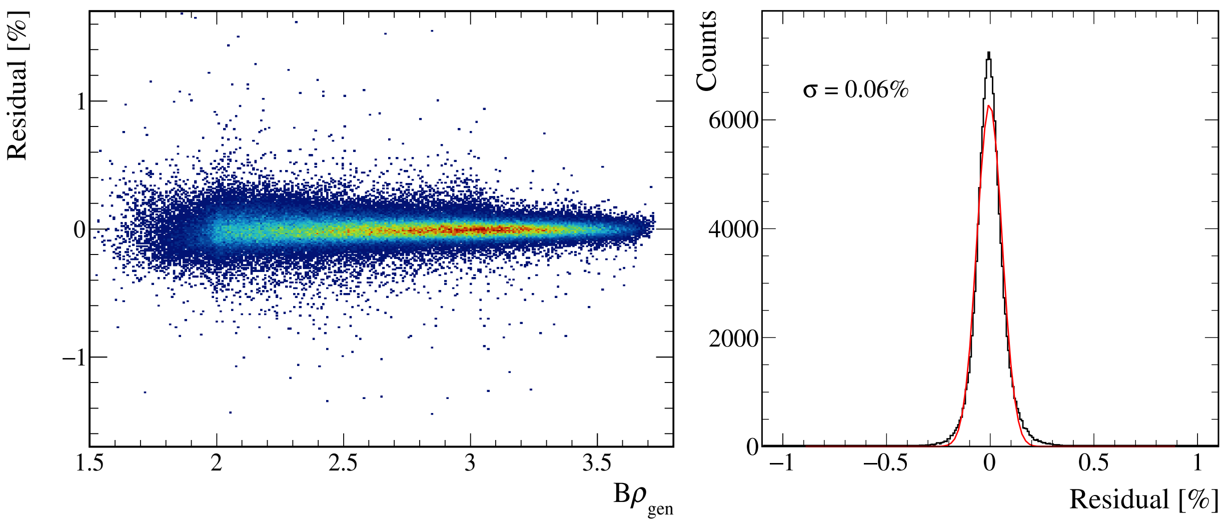

Supplementary Fig. 14: Left: Relative difference between the reconstructed and generated Bρ, $(\mathrm{B}\rho_{\mathrm{mdf}} - \mathrm{B}\rho_{\mathrm{gen}})/\mathrm{B}\rho_{\mathrm{gen}}$ vs. Bρ_{gen}. Right: 1D projection of the relative difference. The width of the distribution represents the tracking precision obtained from the MDF (not including detector resolutions).

In addition, the three-momentum vector of the missing-momentum gives access to the intrinsic momentum of the alpha cluster inside ^{8}He nucleus

$$\boldsymbol{P}_{^4\mathrm{He},int} = -\boldsymbol{P}_{miss}. \tag{7}$$

The momenta of the charged particles are measured in the laboratory frame relative to the beam direction. Lorentz transformation from the laboratory frame into ^{8}He rest frame is applied to obtain the intrinsic momentum distribution of the alpha particle.

Similarly, for the ^{6}He measurement, we replace $\bar{P}_{^8\mathrm{He}} \to \bar{P}_{^6\mathrm{He}}$, $\bar{P}_{4n} \to \bar{P}_{2n}$, and $4 \cdot m_n \to 2 \cdot m_n$. Since we will compare the data to a quasi-elastic simulation, in the following we first describe the simulation procedure, which in general is similar for ^{8}He and ^{6}He, with mainly one but essential difference, which is the expected energy distribution for the $4n$ and $2n$ systems. Then we focus of the measured quasi-elastic events for both ^{8}He and ^{6}He.

4.1 Quasi-elastic simulation

The kinematical code for quasi-elastic reactions was developed by Chulkov *et al.* [6], and we adapt the procedure to calculate the kinematics for the reaction of interest $^8\mathrm{He} + p \to p + ^4\mathrm{He} + 4n$, and similarly for ^{6}He. The ^{8}He is treated as an alpha core plus four neutrons $\boldsymbol{P}_{^8\mathrm{He}} = \boldsymbol{P}_{^4\mathrm{He}} + \boldsymbol{P}_{4n}$. The internal momentum of the alpha core is introduced as Gaussian-shaped distributions in all three directions, with a FWHM of 134 MeV/c, as determined from experiment in Ref. [6]. In the same experiment the motion of the ^{4}He core in ^{6}He was measured as well with a FWHM of 75 MeV/c. The momentum of the residual $4n$ system balances the alpha-core momentum $\boldsymbol{P}_{4n} = -\boldsymbol{P}_{^4\mathrm{He}}$, as we consider here all the quantities in the ^{8}He rest frame. The off-shell mass of the alpha core is determined by

$$M_{\mathrm{off}}^2 = M_{^8\mathrm{He}}^2 + M_{4n}^2 - 2 \cdot M_{^8\mathrm{He}} \cdot \sqrt{M_{4n}^2 + |\boldsymbol{P}_{^4\mathrm{He}}|^2}, \tag{8}$$

where $M_{^8\mathrm{He}}$ is the ^{8}He mass, and M_{4n} is the mass of the four-neutron system. $M_{4n} = 4 \cdot m_n + E_{4n}$, where E_{4n} is the relative energy of the four-neutron system. For ^{8}He we use as an input for E_{4n} the fitted distribution presented in the main text and described in the Methods section, while for ^{6}He the calculated theoretical distribution is used for E_{2n}.

After determination of the energy and momentum of the alpha cluster, the two-body scattering process between the alpha and the target proton is computed in the c.m. frame of the two particles. The $p - ^4$He elastic differential cross section was measured at 156 MeV [7] for the full range of c.m. angles. At large c.m. angles, *i.e.*, backward c.m. scattering, the momentum transfer becomes large enough, such that the proton and alpha are well separated in momentum space, and final-state interactions between the charged particles and neutrons are minimized. We therefore

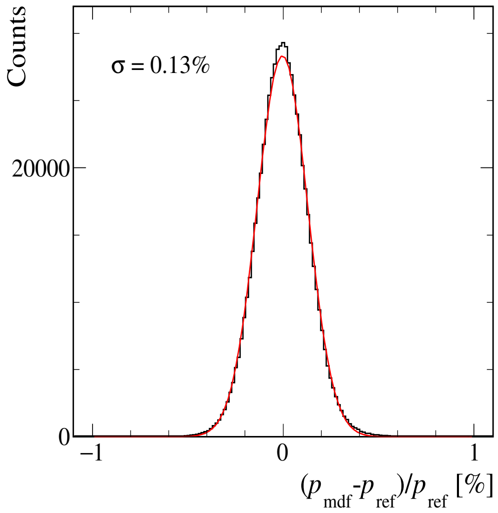

Supplementary Fig. 15: Relative momentum precision obtained using data from proton beam and empty target setup.

consider c.m. angles between $160^\circ < \theta_{\text{c.m.}} < 180^\circ$, where the c.m. angles are generated according to the measured differential distribution in the relevant region multiplied by $\sin(\theta_{\text{c.m.}})$.

The momentum transfer t of the reaction is given by $t = (\bar{P}_{\text{in}} - \bar{P}_{\text{out}})$, where $\bar{P}_{\text{in}}$ is the alpha four-momentum before the scattering, and $\bar{P}_{\text{out}}$ is the one after the scattering process, such that

$$t = M_{\text{off}}^2 + M_{^4\text{He}}^2 - 2 \cdot E_{\text{in}} \cdot E_{\text{out}} + 2 \cdot \boldsymbol{P}_{\text{in}} \cdot \boldsymbol{P}_{\text{out}} \cdot \cos(\theta_{\text{c.m.}}). \tag{9}$$

This equation defines the kinematics of the reaction. For the tracking of the particles in the simulation, momenta are evaluated in the laboratory frame relative to the beam axis. To generate the simulation input sample we use the experimental measured beam profile. The reaction vertex is Gaussian distributed in $x-$ and $y-$direction with a width corresponding to the measured one. In $z-$direction the vertex is uniformly distributed between 0 to 50 mm, the target length. According to the z vertex, the beam energy is corrected for the energy loss in the liquid-hydrogen target. This value is then used in the quasi-elastic code to perform the Lorentz transformation for the charged particles to the laboratory frame after the scattering.

The experimental setup was designed such that the coincident detection of an alpha in HODF and a proton in HODP after passing through the SAMURAI magnet selects large c.m. angles in the range $160^\circ < \theta_{\text{c.m.}} < 180^\circ$ as stated above. As a first check, using the SAMURAI simulator, we generate a sample with wider range $155^\circ < \theta_{\text{c.m.}} < 180^\circ$, shown in Fig. 16 (blue), and compare to the accepted events (red), confirming that the accepted events cover the correct range, and almost no events with c.m. angles below 160° are accepted.

After passing the sample through the simulated experimental setup we request hits in all silicon planes, FDC2, and HODF (HODP) for alpha (proton). To include the detector resolutions, the position and angles in the silicon planes and FDC2 are smeared according to the measured resolutions. The smeared simulated data are then further analyzed exactly the same manner as the experimental data, using the MDF function for momentum reconstruction, reconstructing the reaction vertex from the silicon trackers, and correcting for the energy loss in the liquid-hydrogen target. Similarly, for the incoming beam, the momentum vector is smeared as deduced from the TOF resolution of the SBTs, and the position resolution of the BDCs. Additionally, we include the angular and energy straggling of the incoming beam in the target using ATIMA code [4].

4.2 Events characterization

To characterize the measured quasi-elastic $^{8(6)}\text{He}(p, p^4\text{He})$ events, we look at the kinematic variables measured and extracted from the reaction, and compare them to those obtained from the quasi-elastic simulation, with the different inputs for ^{8}He and ^{6}He. In the following, distributions for simulated data are always normalized according to the number of measured events, and we use the abbreviation QE whenever referring to quasi-elastic.

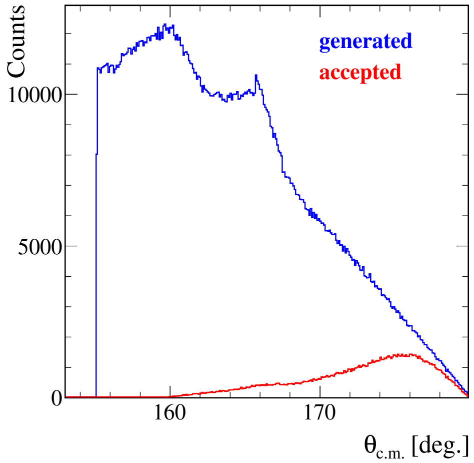

Supplementary Fig. 16: Angular distribution of the c.m. angle for generated (blue) quasi-elastic $p-{}^4$He events and for events accepted (red) by the SAMURAI setup.

We start by extracting the c.m. angle of the QE $p-{}^4$He scattering. As stated, the idea of the measurement was to consider the reaction at backward angles, $160^\circ < \theta_{\mathrm{c.m.}} < 180^\circ$, in order to maximize the momentum transfer between the charged particles and hence minimize final-state interactions between the charged particles and the neutrons. To confirm, Fig. 17 shows the c.m. angular distribution for ^{8}He (left) and ^{6}He (right).

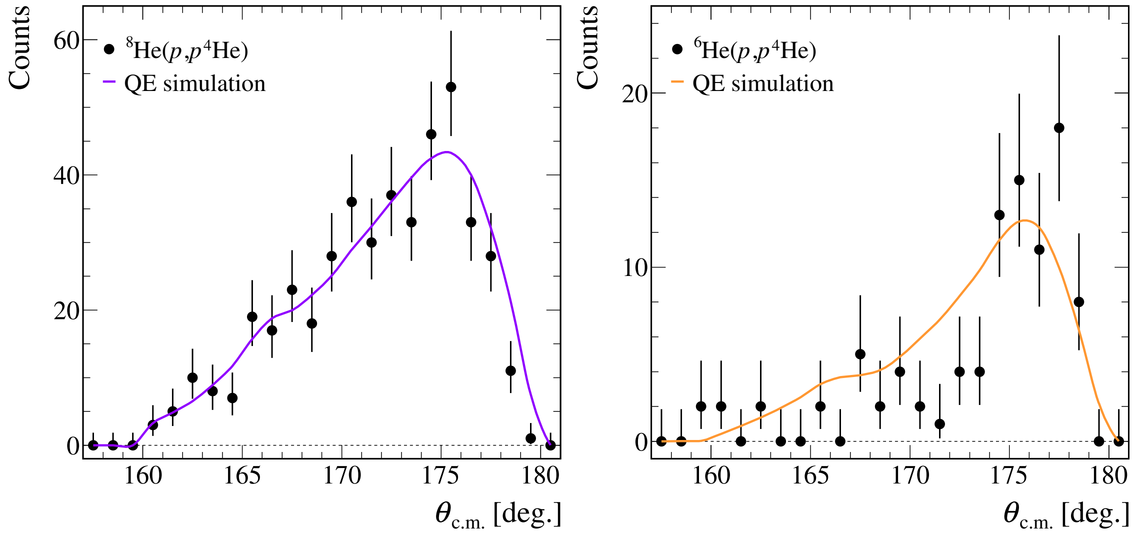

Supplementary Fig. 17: Measured c.m. angular distribution in the QE $p-{}^4$He scattering from ^{8}He (left) and ^{6}He (right) compared to QE simulation (solid curves).

The data show only events with large c.m. angles, as expected, without any contribution from lower c.m. angles, which would increase significantly the probability for final-state interactions between the charged particles and neutrons, and could yield a contamination or distortion of the spectrum due to secondary-scattering processes.

Large c.m. angles correspond to reactions with large momentum transfer. Figure 18 shows the invariant energy- and momentum-transfer Mandelstam variables defined by the measured proton and alpha four-momenta, $\bar{P}_p$ and $\bar{P}_{^4\mathrm{He}}$

$$u = (\bar{P}_{^4\mathrm{He}} - \bar{P}_{p(\mathrm{tgt})})^2, \quad t = (\bar{P}_p - \bar{P}_{p(\mathrm{tgt})})^2, \quad s = (\bar{P}_p + \bar{P}_{^4\mathrm{He}})^2, \tag{10}$$

where $\bar{P}_{p(\mathrm{tgt})}$ is the momentum of the target proton. Measured $^{8(6)}$He distributions show overall a good agreement with the simulated distributions. This provides further support to the cleanliness of our selected event sample.

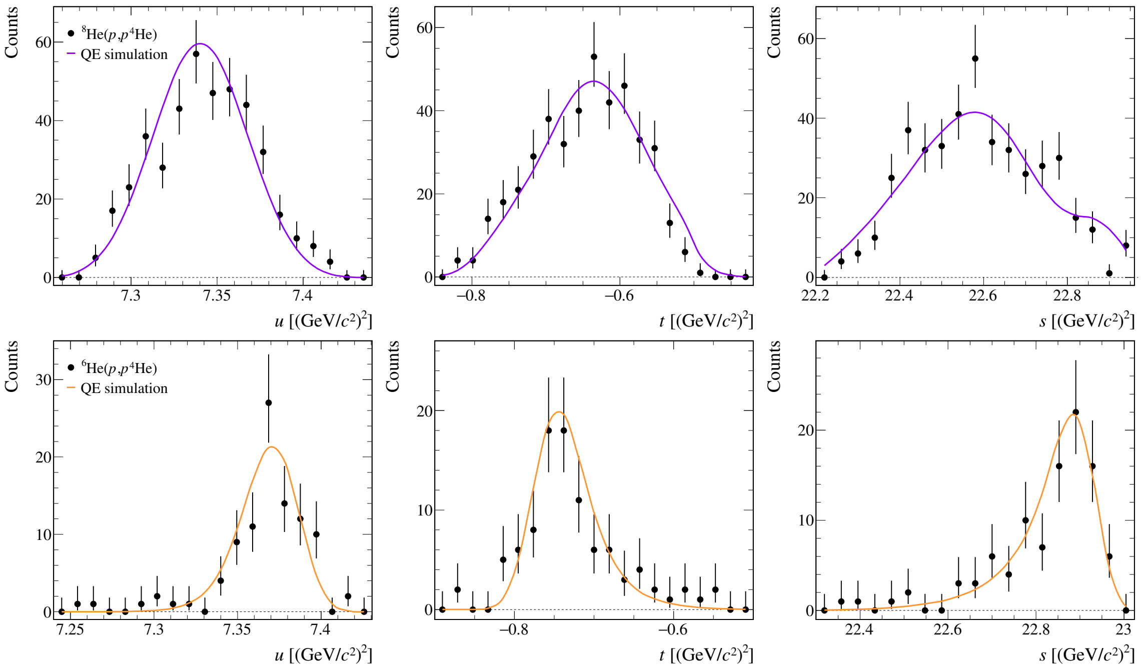

Supplementary Fig. 18: Distributions of Mandelstam variables as measured in the QE $p-{}^4$He reaction from ^{8}He (top) and ^{6}He (bottom) compared to QE simulation (solid curves).

As a result of the large momentum transferred between the proton and ^{4}He, the knocked-out alpha is slowed down from its initial momentum, *i.e.*, with the incoming beam velocity, while the proton, which was at rest in the initial state, becomes the fastest particle. Figure 19 shows the measured momenta of the knocked-out alpha, scattered proton, and for comparison the incoming beam momentum for ^{6}He sample, where the distributions for ^{8}He are shown in Fig. 1 of the main paper. It can be seen that the distributions follow those expected from the QE simulation, and do not exceed their limits.

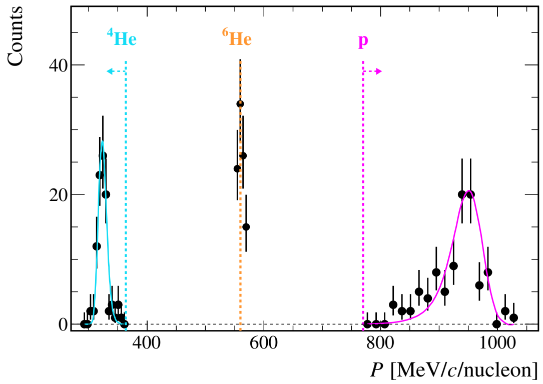

Supplementary Fig. 19: Measured momentum of the knocked-out alpha and scattered proton after the QE reaction for ^{6}He sample (black symbols) together with QE simulation (solid curves). The cyan (magenta) dashed line represents the upper (lower) limit of the alpha (proton) momentum expected from QE simulation. Also shown is the momentum of incoming ^{6}He, where the orange dashed line indicates the central beam momentum.

Next we consider the angular distributions of the two particles. Figure 20 shows the polar scattering angles of the alpha and proton. Both agree with the expected angular distributions from the QE simulation and no events are found at the edges of the setup's acceptance.

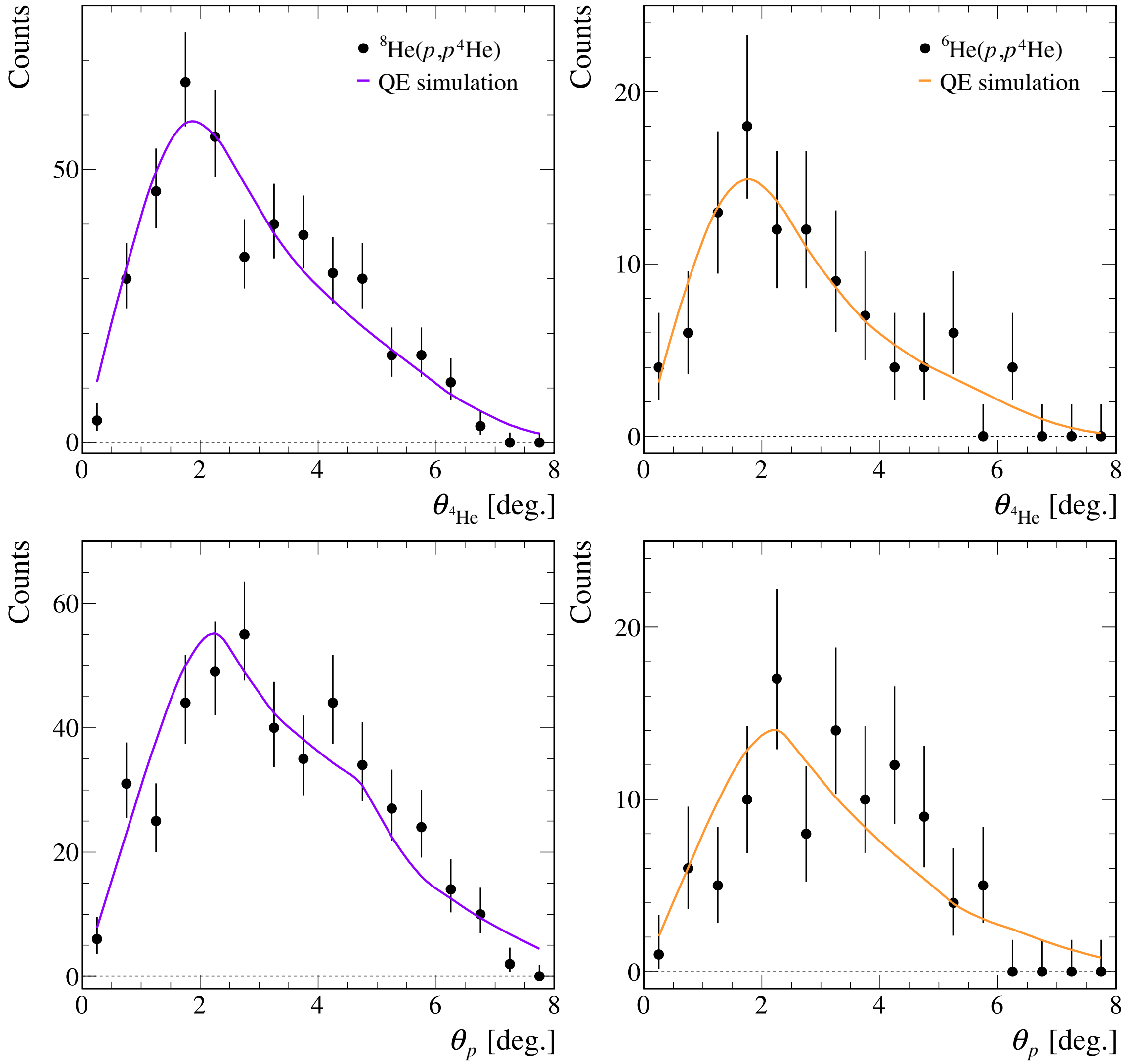

Supplementary Fig. 20: Polar scattering angles for the knocked-out alpha (top) and scattered proton (bottom) from ^{8}He (left) and ^{6}He (right) together with QE simulation (solid curves).

The opening angle between the proton and alpha is calculated using the scalar product of their three-momenta vectors

$$\text{Opening Angle} = \arccos\left(\sin\theta_{^4\mathrm{He}}\sin\theta_p\cos(\phi_{^4\mathrm{He}} - \phi_p) + \cos\theta_{^4\mathrm{He}}\cos\theta_p\right), \quad (11)$$

where $\phi_{^4\mathrm{He}(p)}$ is the azimuth angle of the alpha (proton). Figure 21 (top) shows the opening angle between the proton and alpha, compared to the simulated QE distribution. The distributions are peaked at small angles for both $^{8(6)}$He as expected. Very small angles are suppressed since they cannot be resolved by the silicon trackers. Since the energy deposition of the proton and alpha in the silicon tracker is very different, they are separated by several strips, which results in minimum angular separation of about 0.5°. Also shown in Fig. 21 (bottom) the azimuth angle $\Delta\phi = |\phi_{^4\mathrm{He}} - \phi_p|$, where for $\Delta\phi > 180°$, we take $\Delta\phi \to 360° - \Delta\phi$. It approaches the maximum at 180° with a tail towards smaller angles resulting from the intrinsic momentum of the alpha in ^{8,6}He, with a larger tail for ^{8}He due to a wider motion of the alpha [6].

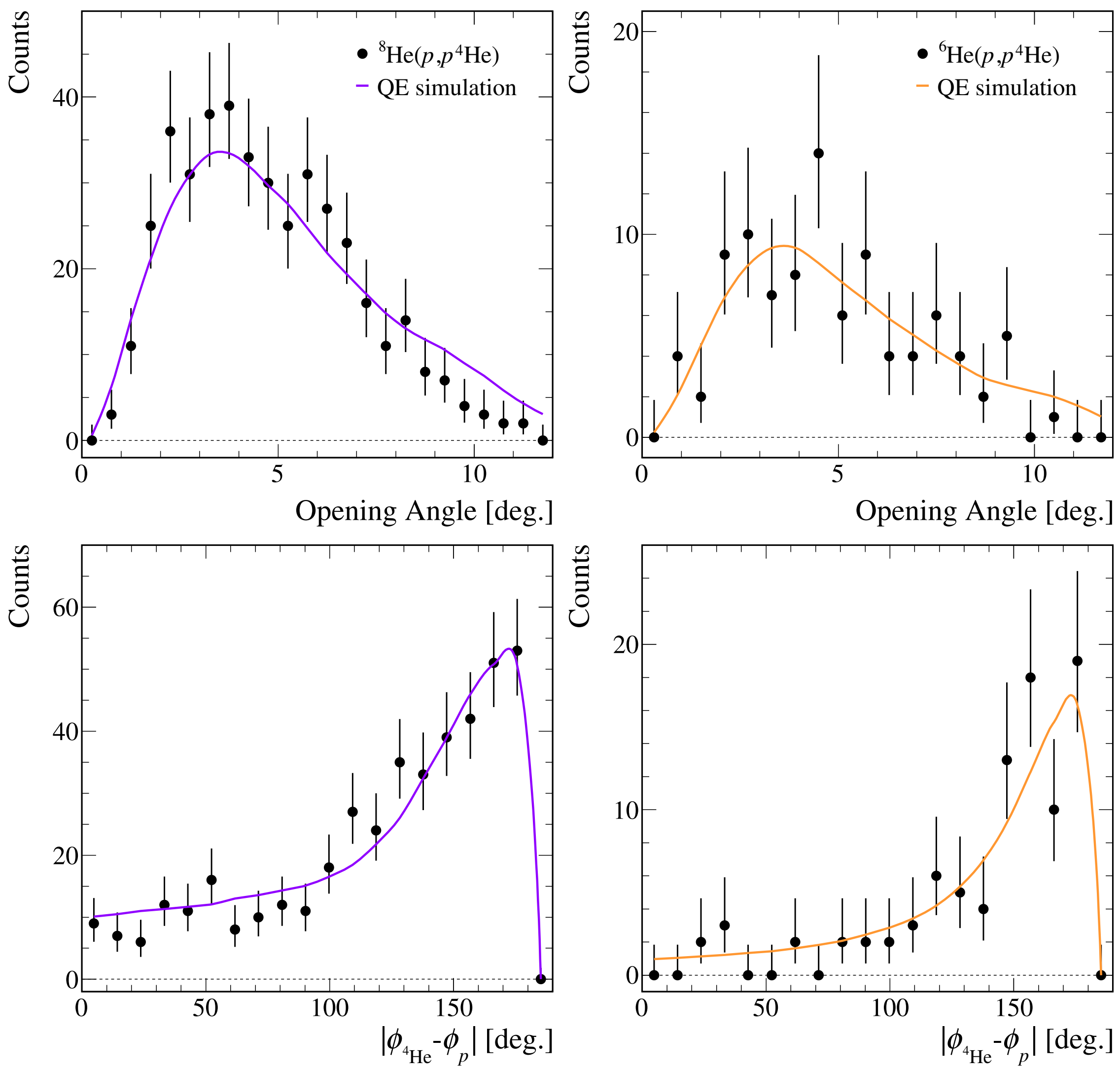

Supplementary Fig. 21: Opening angle between the knocked-out alpha and scattered proton (top) and azimuth angle $\Delta\phi = |\phi_{^4\mathrm{He}} - \phi_p|$ (bottom) from ^{8}He (left) and ^{6}He (right) together with QE simulation (solid curves).

Finally, as described above, from the three-vector of the missing-momentum, Eq. 7, we gain access to the intrinsic momentum of the alpha core inside $^{8(6)}$He. Figure 22 shows the total missing-momentum for ^{8}He (left) and ^{6}He (right) samples. The distribution observed for ^{8}He is wider and centered at larger momentum than that of ^{6}He. This is in agreement with the previously measured distributions [6] which yielded in FWHM of 134 MeV/c and 75 MeV/c, respectively, taken as an input for the QE simulations. Figure 23 shows in addition the different components of the missing-momentum.

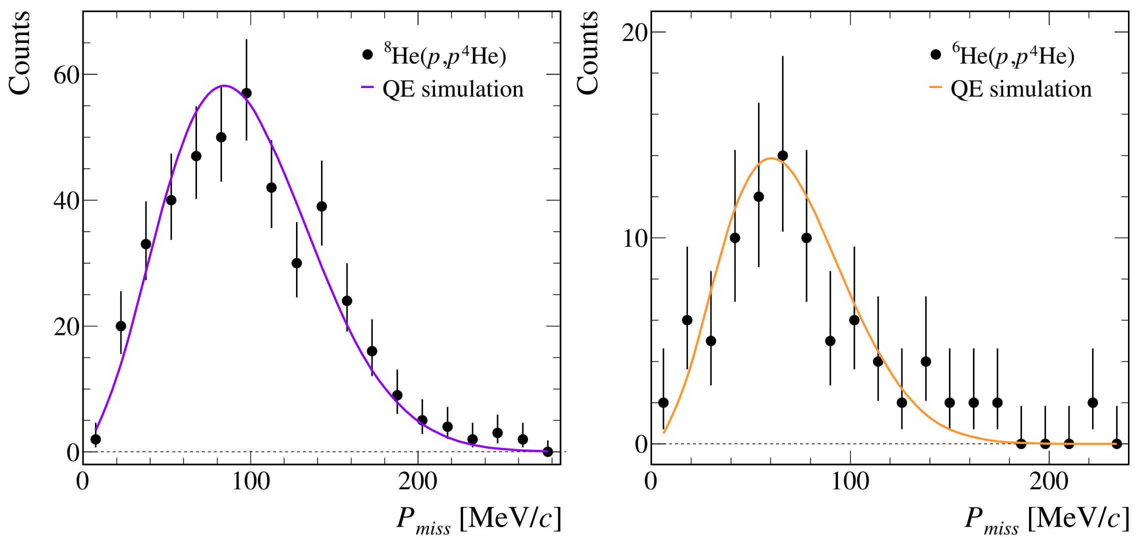

Supplementary Fig. 22: Total missing-momentum, corresponds to the intrinsic motion of the alpha core in ^{8}He (left) and ^{6}He (right) together with QE simulation (solid curves), where we used Gaussian distributions with FWHM of 134 MeV/c and 75 MeV/c for all three directions, respectively.

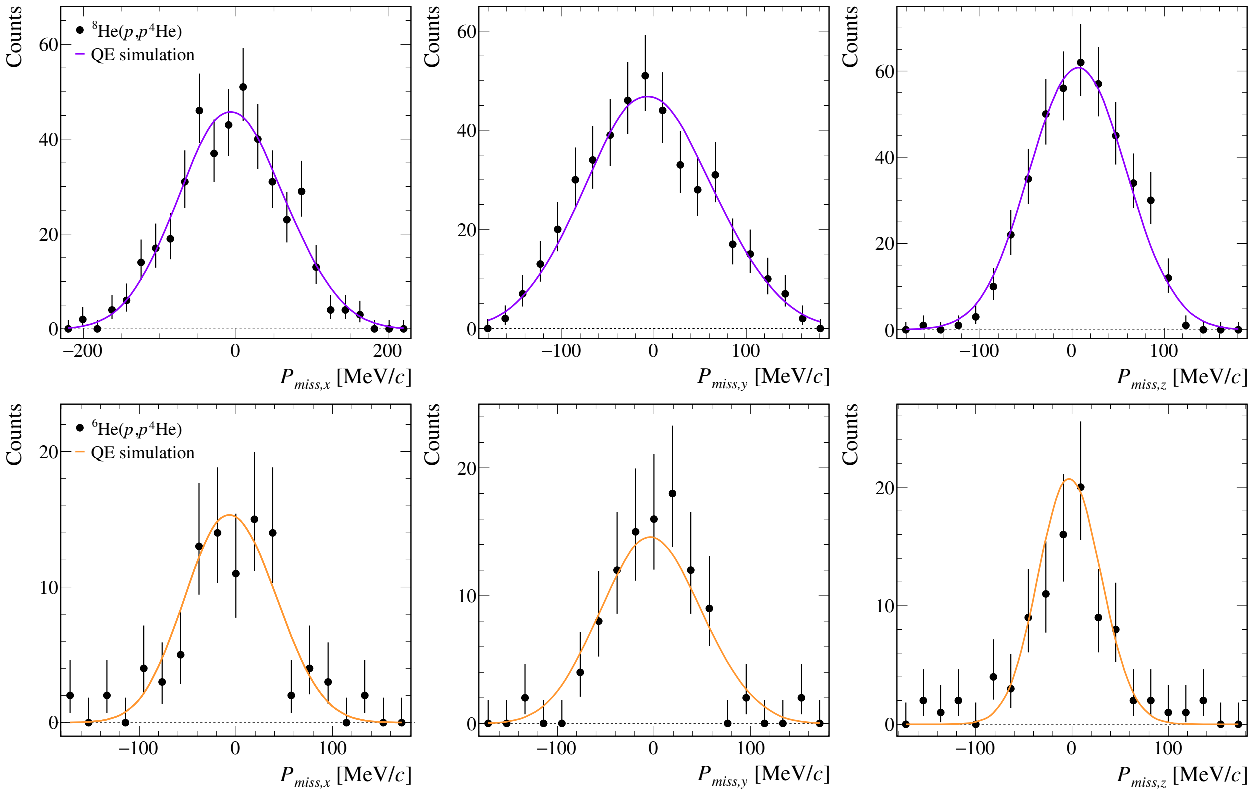

Supplementary Fig. 23: Missing-momentum components in x, y, z directions for ^{8}He (top) and ^{6}He (bottom) samples together with QE simulation (solid curves).

4.3 Statistics estimates

Below we compare the measured statistics for $^8\text{He}(p,p^4\text{He})$ (422 events) and $^6\text{He}(p,p^4\text{He})$ (99 events), to that expected from rate estimation. To do so we need to take into account several factors:

- N_{beam} : The number of incoming $^{8(6)}$He ions is evaluated by the number of $^{8(6)}$He events which pass the incoming identification cuts presented in Section 2, where we take into account the down-scaling factors of the different triggers discussed in Section 1. This results in $N_{beam} = 24.9 \cdot 10^9$ events for ^{8}He and $N_{beam} = 5.4 \cdot 10^9$ events for ^{6}He.
- σ : As discussed in this section, the $p - {}^4\text{He}$ elastic differential cross section was measured [7]. For large c.m. angles in the range $160° < \theta_{c.m.} < 180°$, the integrated cross section equals $6 \cdot 10^{-3}$ mb.
- Acc: The cross section as shown by the blue histogram in Fig. 16 (where we consider only the range of $160° < \theta_{c.m.} < 180°$) is reduced due to the acceptance of the setup as shown by the red histogram in Fig. 16. The overall acceptance in this range equals 10%. This is mainly due to the angular acceptance of the SAMURAI setup which corresponds roughly to $\pm 2.5°$ and $\pm 5°$ in vertical and horizontal directions, respectively.
- t: For a 5 cm liquid hydrogen target with density of 0.07 g/cm^3 the thickness equals 0.35 g/cm^2.
- ε: The detection efficiency for $^{8(6)}\text{He}(p,p^4\text{He})$ events is estimated by $\varepsilon = \varepsilon_{BDC} \times \varepsilon_{SI} \times \varepsilon_{FDC2} = 0.94 \times 0.67 \times 0.49 = 0.31$, as extracted in Sections 2 and 3.

Combining these factors, the number of expected reactions N_{reac} is estimated as

$$N_{reac} = N_{beam} \times \sigma(\text{b}) \times \text{Acc} \times t(\text{g/cm}^2) \times 0.6(\text{Avogadro})/\text{A} \times \varepsilon = \tag{12}$$

$$= 24.9 \cdot 10^9 \times 6 \cdot 10^{-6} \times 0.1 \times 0.35 \times 0.6/1 \times 0.31 = 900 \text{ events}$$

for ^{8}He, and

$$= 5.4 \cdot 10^9 \times 6 \cdot 10^{-6} \times 0.1 \times 0.35 \times 0.6/1 \times 0.31 = 200 \text{ events}$$

for ^{6}He. Both values have the same order of magnitude as the measured events of 422 and 99, respectively.

It should be noted that we assumed here a detection efficiency of 100% for the HODs, which is not necessarily the case for the fast protons. In addition, as stated in Section 3 the detection efficiencies extracted for the silicon trackers and FDC2 are only an upper limit.

Finally, we expect for flux loss due to the attenuation of the incoming $^{8(6)}$He as it travels through the target, which we approximate as $\sim 10\%$ for both cases.

We conclude that the observed number of events for the $^{8(6)}\text{He}(p,p^4\text{He})$ reactions is close to the expectation. The lower statistics for the $^6\text{He}(p,p^4\text{He})$ is due to the shorter beam-time with this setting. However, the statistics in the low-energy (peak) region is as expected from the theoretical estimation comparable.

4.4 Secondary reactions

As stated in the main text and the Methods section, events with a fast proton and a slow alpha in the final state can result from background coming from competing processes. In the Methods section we identified four possible contributions from secondary reactions: ^{4}He production, single-neutron knockout, ^{6}He knockout, and inelastic excitation of ^{8}He. The total contribution of these events to the missing-mass spectrum is presented by the green curve in Fig. 3 of the main text. The shape of the background was estimated by simulating individually each one of the possible two-step reactions, and weighting them according to the measured cross sections.

The starting point of simulating any two-step process is always the same, incoming ^{8}He with the same beam profile as used in the QE simulation, and a first interaction vertex z_1 uniformly distributed between 0 to 50 mm. The second interaction point then occurs at z_2, which is distributed uniformly between z_1 to 50 mm. The missing-mass spectrum is reconstructed in the exact same way as in the data, *i.e.*, from the momentum of incoming ^{8}He, and the momenta of the proton and alpha produced after the second interaction.

Below we detail the exact simulation procedures used for the different processes:

- ^{4}He production: In the first interaction point z_1, ^{4}He is produced by breakup of ^{8}He into ^{4}He and 4n with a separation energy of 3.1 MeV. In the ^{8}He rest frame, the ^{4}He recoils according to its internal momentum, $(\boldsymbol{P}_{^4\mathrm{He}}, \sqrt{M^2_{^4\mathrm{He}} + |\boldsymbol{P}_{^4\mathrm{He}}|^2})$, and is balanced by the 4n system which carries momentum $(-\boldsymbol{P}_{^4\mathrm{He}}, \sqrt{M^2_{4n} + |\boldsymbol{P}_{^4\mathrm{He}}|^2})$ (with $E_{4n} = 0$). The internal momentum is introduced similarly as in the QE simulation, Gaussian-shaped distributions in all three directions with a FWHM of 134 MeV/c [6]. The energy-loss of ^{4}He between the two interaction points, $z_2 - z_1$, is considered, and in the second interaction point we generate the $p - {}^4$He QE backward c.m. scattering according to the momentum of the ^{4}He in that point.
  The resulting simulated missing-mass spectrum is shown by the blue curve in Fig. 24, which represents the reconstructed spectrum, taking into account the experimental response, acceptance and detector resolutions, as well as the reconstruction algorithms of the two fragments. The contribution from this two-step process occurs at the negative region of the missing-mass spectrum with a threshold at the binding energy of -3.1 MeV, and it extends to more negative values due to the internal motion of the ^{4}He.
  The inclusive cross section of $^8\mathrm{He} + {}^{12}\mathrm{C} \rightarrow {}^4\mathrm{He}$ was measured to be 95 ± 5 mb [8]. We scale by a factor of 1/2 to estimate the cross section for the hydrogen target, leading to $\sigma = 48 \pm 5$ mb. The relative contribution to the number of measured events is evaluated as 0.5%.

- Single-neutron knockout: In the first interaction point we consider the $^8\mathrm{He}(p, pn)^7\mathrm{He}$ reaction with a separation energy of 2.5 MeV. In the ^{8}He rest frame, $\boldsymbol{P}_{1n} = -\boldsymbol{P}_{^7\mathrm{He}}$, where the single-neutron momentum distribution is taken as Gaussian-shaped distributions in all three directions with a FWHM of 188 MeV/c [9]. After the neutron knockout the resonant ^{7}He state recoils according to the intrinsic momentum, and decays directly to $^6\mathrm{He} + n$. We modeled the $^6\mathrm{He} + n$ from the phase-space decay of the ^{7}He resonance according to its measured energy and width of 0.385 MeV and 0.193 MeV, respectively [10]. The energy-loss of ^{6}He between the two interaction points, $z_2 - z_1$,is considered, and in the second interaction point we generate the $^6\mathrm{He}(p, p^4\mathrm{He})2n$ QE backward c.m. scattering according to the momentum of ^{6}He in that point, using the calculated distribution of E_{2n} as described in the Methods section. The resulting simulated missing-mass spectrum is shown by the black curve in Fig. 24.
  To estimate the cross section we scaled the measured cross sections in [10], which yields $\sigma = 59 \pm 9$ mb. The relative contribution to the number of measured events is evaluated as 0.9%.

- ^{6}He knockout: In the first interaction point we generate the $^8\mathrm{He}(p, p^6\mathrm{He})2n$ QE scattering in the full range of c.m. angles with a separation energy of 2.1 MeV. In the ^{8}He rest frame $\boldsymbol{P}_{^6\mathrm{He}} = -\boldsymbol{P}_{2n}$, where the ^{6}He momentum distribution is taken as Gaussian-shaped distributions in all three directions with a FWHM of 82 MeV/c [6]. After the production of ^{6}He, we repeat the same procedure as described above for the second interaction, the single-neutron knockout. The resulting simulated missing-mass spectrum is shown by the red curve in Fig. 24. We evaluated the cross section being similar to $p - {}^4$He cross section [6] and adopted a value of $\sigma = 91.8$ mb [7]. The relative contribution to the number of measured events is evaluated as 0.96%.

- Inelastic excitation of ^{8}He : In the first interaction point ^{6}He is produced by breakup of $^8\mathrm{He}^*$ followed from the $^8\mathrm{He}(p, p)^8\mathrm{He}^*$ reaction. In such a case $M_{^8\mathrm{He}} + E_x(3.1\ \mathrm{MeV}) \rightarrow {}^6\mathrm{He} + 2n$. In the ^{8}He rest frame, the ^{6}He recoils according to its internal momentum (FWHM 82 MeV/c), which is treated in the same way as for the other reactions where ^{6}He is produced. The resulting simulated missing-mass spectrum is shown by the magenta curve in Fig. 24.
  To estimate the cross section, again, we scaled the measured cross section [10], which yields $\sigma = 11 \pm 2$ mb. The relative contribution to the number of measured events is evaluated as 0.2%.

Overall the expected background contribution from two-step reactions is about 2.6%, and is shown by the dashed green curve in Fig. 24, where we summed the different processes according to their relative contributions. The green band represents an uncertainty obtained by considering a factor of two difference for the cross sections used for the estimates, which accounts for possible uncertainties due to the need of scaling the measured cross sections to the reaction of interest in this work.

For the benchmark measurement with ^{6}He only one secondary reaction can contribute to the background, ^{4}He production. Similarly to the ^{8}He case, we simulate this two-step reaction as follows: in the first interaction point ^{4}He is produced by breakup of ^{6}He into ^{4}He and 2n with a separation energy of 0.975 MeV. In the ^{6}He rest frame, the ^{4}He recoils according to its internal momentum and is balanced by the 2n system. The internal momentum is taken as

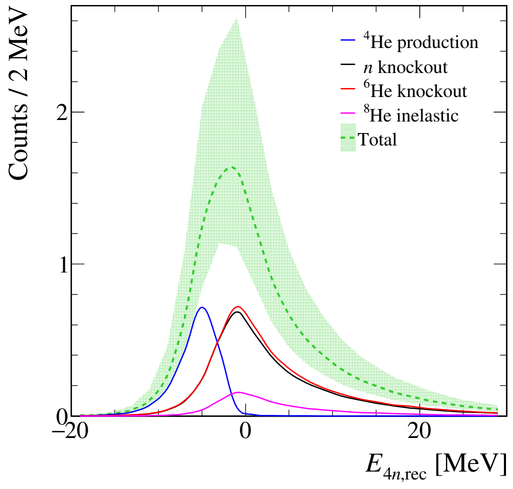

Supplementary Fig. 24: Simulated missing-mass spectra expected from two-step reactions: ^{4}He production (blue), neutron knockout (black), ^{6}He knockout (red), and inelastic excitation of ^{8}He (magenta). The dashed green curve represents the total contribution together with an uncertainty (green band) obtained by considering a factor of two difference on the cross sections used to estimate the different contributions.

Gaussian shaped distribution in all three directions with a FWHM of 75 MeV/c [6]. The energy-loss of ^{4}He between the two interaction points is considered, and in the second interaction point we generate the $p-{}^{4}$He QE backward c.m. scattering.
The inclusive cross section of $^{6}\mathrm{He}+{}^{12}\mathrm{C}\rightarrow{}^{4}\mathrm{He}$ was measured to be 189 ± 14 mb [8]. We scale by a factor of 1/2 to estimate the cross section for the hydrogen target, leading to $\sigma=95\pm7$ mb. The relative contribution to the number of measured events is evaluated as 1%. The resulting simulated missing-mass spectrum is shown by the green curve in Fig. 25.

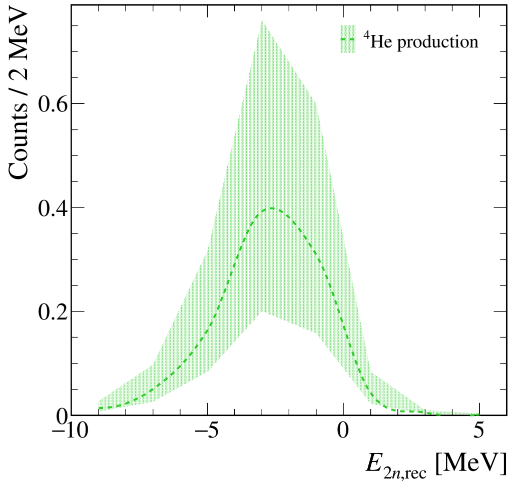

Supplementary Fig. 25: Simulated missing-mass spectrum for ^{6}He benchmark measurement expected from two-step reaction where ^{4}He is produced in a first step.

4.5 Neutron trigger

Although the neutron detection is out of the scope of the analysis discussed, for completeness we considered the neutron trigger for our selected QE $^{8(6)}$He(p, p^4He) events. As described in the Methods section another experiment was performed within the same experimental campaign as the experiment presented here, studying the low-energy dipole response of ^{6,8}He. A detailed analysis of the neutrons involved in these reactions was carried out, including measurement of four neutrons in coincidence [10]. We adapt the same procedures to look for coincident events with one or more neutrons.

Figure 26 shows the fraction of events with no detected neutrons, one neutron, and two neutrons in coincidence, for ^{8}He (left) and ^{6}He (right) QE samples. For ^{8}He due to the low statistics of our sample we observed zero events with three or four neutrons in coincidence.

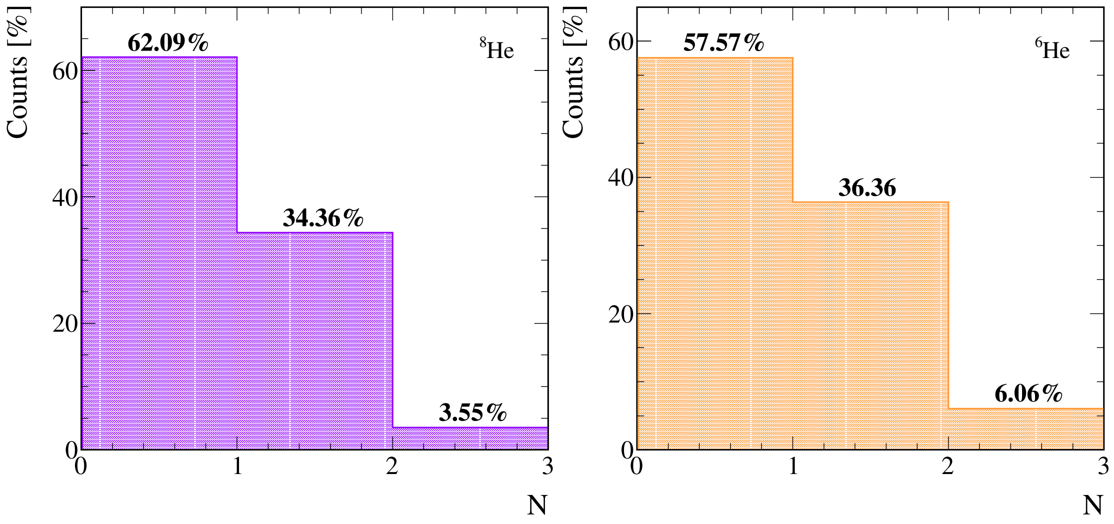

Supplementary Fig. 26: Fraction of neutrons detected in coincidence with the QE events from ^{8}He (left) and ^{6}He (right).

To check if the observed fractions are realistic we take into account the neutron detection efficiency and acceptance using the SAMURAI simulator. We generate our QE ^{8,6}He(p, p^4He) events, and modeled the individual neutrons from a phase-space decay according to the energy of the four- (two-) neutron system $E_{4(2)n}$. After passing the sample through the simulated experimental setup, the efficiency for detecting N neutrons is defined by the fraction of events with N reconstructed neutrons relative to the number of reconstructed $p-{}^4$He events. Figure 27 (top) shows the detection efficiency as a function of the energy of the two-neutron system, reconstructed using the missing-mass *i.e.*, from the charged particles, for reconstructing exactly one or two neutrons from ^{6}He. Similarly, Fig. 27 (bottom) shows the efficiency for detecting one, two and three neutrons from ^{8}He. Using the resulted efficiencies, we evaluate the fraction of expected events with detected neutrons. For ^{6}He this corresponds to 33% with one neutron detected and 5% with two neutrons. For ^{8}He a fraction of 39% with one neutron, 5% with two neutrons, and less than 0.5% with three neutrons. Overall, these are consistent with the observed fractions shown in Fig. 26. Note that a fraction of $\sim 0.5\%$ with three neutrons will correspond to 2 events, and is therefore consistent with the zero measured events.

For events with one detected neutron, the statistics allow to look at its velocity. In the rest frame of $^{8(6)}$He it is expected to have small velocity that is modified due to the energy of the four- (two-) neutron system. This is demonstrated in Fig. 28 which shows the measured β_n distribution for such cases for ^{8}He (left) and ^{6}He (right) samples, together with the expected distribution from QE simulation.

Additionally, we consider the polar scattering angle of the neutron, in the laboratory frame, for $^{8(6)}$He sample, shown in Fig. 29, together with the QE simulation. In particular, for ^{8}He we look separately at the two components observed in the missing-mass spectrum: the continuum, $E_{4n} > 6$ MeV (full symbols and solid curve), and the resonance peak region, $-2 < E_{4n} < 6$ MeV (open symbols and dashed curve). For the resonance peak region we expect that the neutrons will be more forward focused, *i.e.*, smaller scattering angles, whereas for the continuum they are expected to

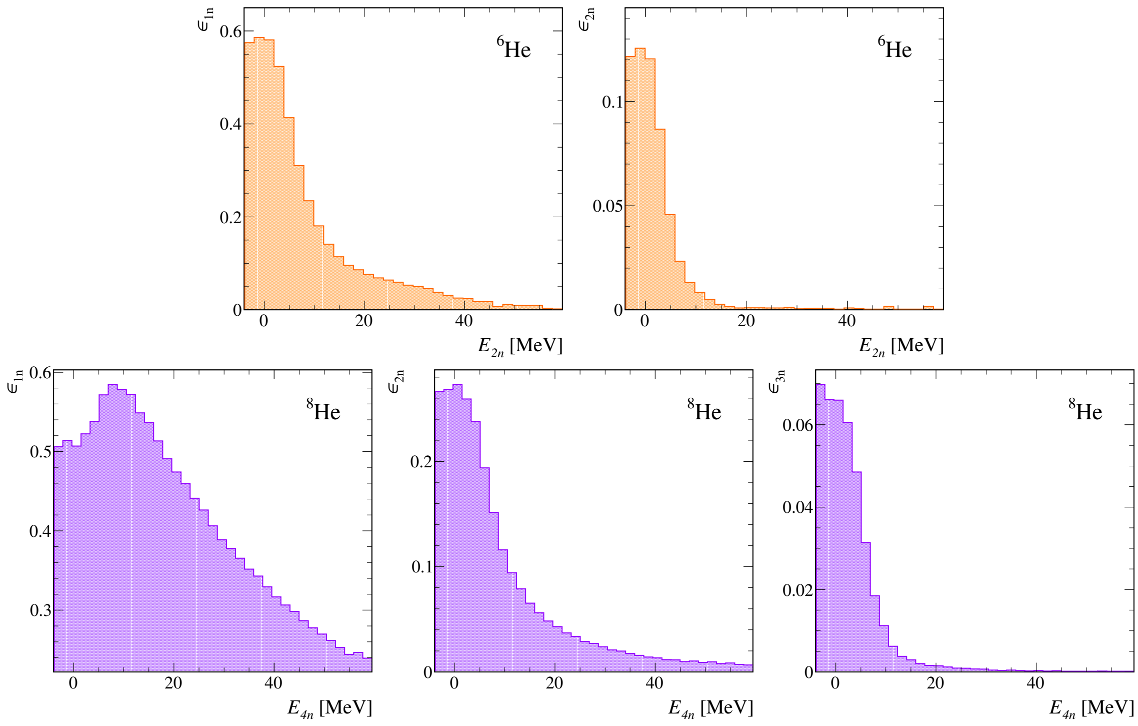

Supplementary Fig. 27: Top: Detection efficiency of one (left) and two (right) neutrons after QE $p - {}^4$He reaction from ^{6}He as a function of the energy of the two-neutron system. As the energy is reconstructed from the momenta of the charged particles, due to the resolution it is smeared to negative values. Bottom: Same but for ^{8}He, including three neutrons.

have a wider distribution expanded towards larger angles. Although the statistics for the resonance peak is very small, a clear difference is observed between the two components, in good agreement with the QE simulation.

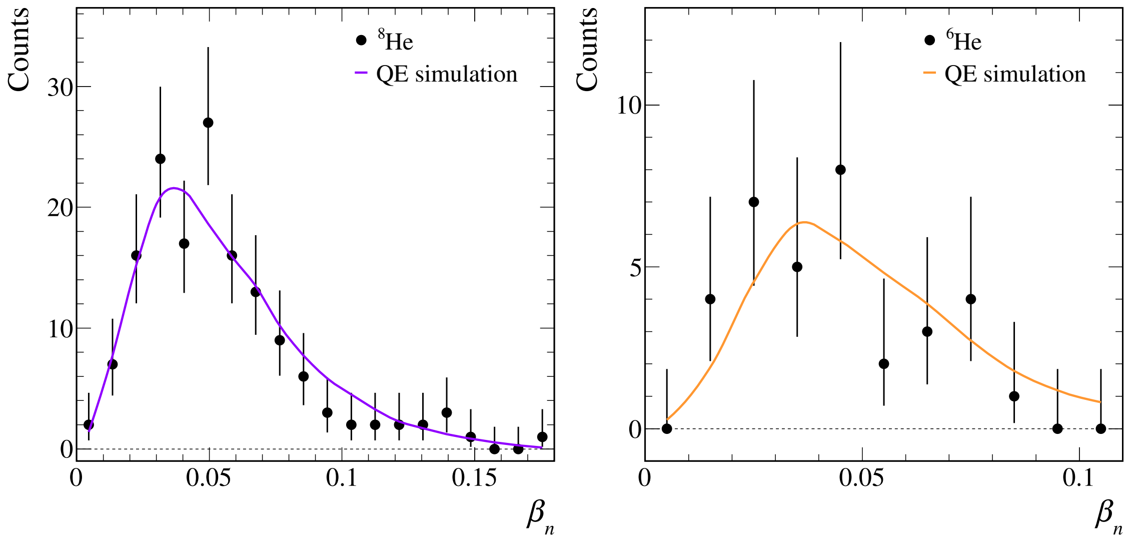

Supplementary Fig. 28: Neutron velocity at the rest frame of ^{8}He (left) and ^{6}He (right) for QE events with one detected neutron. The solid curves represent the expected distribution from QE simulation, *i.e.*, QE scattering followed by a phase-space decay of the neutron system.

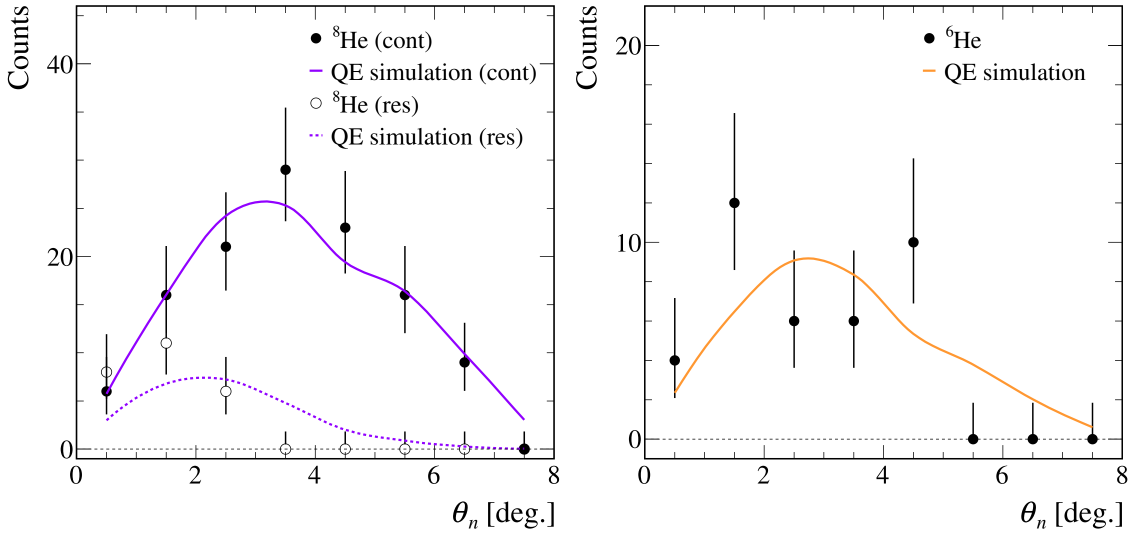

Supplementary Fig. 29: Polar scattering angle of the neutron for QE ^{8}He (left) and ^{6}He (right) events with one detected neutron. The curves represent the expected distribution from QE simulation, *i.e.*, QE scattering followed by a phase-space decay of the neutron system. For ^{8}He we consider the two components in the missing-mass spectrum separately: the continuum (full symbols and solid curves) and the resonance peak (open symbols and dashed curve).

References

[1] T. Kobayashi et al. “SAMURAI spectrometer for RI beam experiments”. In: *Nucl. Instrum. Meth. B* 317 (2013), pp. 294–304.

[2] A. Obertelli et al. “MINOS: A vertex tracker coupled to a thick liquid-hydrogen target for in-beam spectroscopy of exotic nuclei”. In: *Eur. Phy. J. A* 50 (2014), p. 8.

[3] Geant4 simulation. URL: https://geant4.web.cern.ch.

[4] ATIMA GSI. URL: https://web-docs.gsi.de./~weick/atima.

[5] ROOT Cern: Multi-Dimensional fit. URL: https://root.cern.ch/doc/master/classTMultiDimFit.html.

[6] L. V. Chulkov et al. “Quasi-free scattering with ^{6,8}He beams”. In: *Nucl. Phys. A* 759 (2005), p. 43.

[7] V. Comparat et al. “Elastic proton scattering on ^{4}He at 156 MeV”. In: *Phys. Rev. C* 12 (1975), p. 251.

[8] Tanihata I. et al. “Revelation of thick neutron skins in nuclei”. In: *Phys. Lett. B* 289 (1992), p. 261.

[9] Aksyutina Yu. et al. “Momentum profile analysis in one-neutron knockout from Borremean nuclei”. In: *Phys. Lett. B* 718 (2013), p. 1309.

[10] C. Lehr. “Low-energy dipole response of the halo nuclei ^{6,8}He”. PhD thesis. TU Darmstadt, 2022. URL: https://tuprints.ulb.tu-darmstadt.de/20267/.
